# Preferences for Attributes of Initial COVID-19 Diagnosis in the United States and China During the Pandemic: Discrete Choice Experiment With Propensity Score Matching

**DOI:** 10.2196/37422

**Published:** 2022-08-16

**Authors:** Yimin Zhang, Taoran Liu, Zonglin He, Sze Ngai Chan, Babatunde Akinwunmi, Jian Huang, Tak-Hap Wong, Casper J P Zhang, Wai-Kit Ming

**Affiliations:** 1 School of Medicine Jinan University Guangzhou China; 2 Department of Infectious Diseases and Public Health City University of Hong Kong Hong Kong China; 3 Division of Life Science Hong Kong University of Science and Technology Hong Kong Hong Kong; 4 Department of Obstetrics and Gynaecology First Affiliated Hospital of Jinan University Guangzhou China; 5 Department of Obstetrics and Gynecology Brigham and Women's Hospital Boston, MA United States; 6 Center for Genomic Medicine Massachusetts General Hospital Boston, MA United States; 7 Singapore Institute for Clinical Sciences Agency for Science, Technology and Research Singapore Singapore; 8 School of Public Health University of Hong Kong Hong Kong China

**Keywords:** COVID-19, public health, discrete choice experiment, patient preference, propensity score matching, patients with fever

## Abstract

**Background:**

China and the United States play critical leading roles in the global effort to contain the COVID-19 virus. Therefore, their population’s preferences for initial diagnosis were compared to provide policy and clinical insights.

**Objective:**

We aim to quantify and compare the public’s preferences for medical management of fever and the attributes of initial diagnosis in the case of presenting symptoms during the COVID-19 pandemic in China and the United States.

**Methods:**

We conducted a cross-sectional study from January to March 2021 in China and the United States using an online discrete choice experiment (DCE) questionnaire distributed through Amazon Mechanical Turk (MTurk; in the United States) and recruited volunteers (in China). Propensity score matching (PSM) was used to match the 2 groups of respondents from China and the United States to minimize confounding effects. In addition, the respondents’ preferences for different diagnosis options were evaluated using a mixed logit model (MXL) and latent class models (LCMs). Moreover, demographic data were collected and compared using the chi-square test, Fisher test, and Mann-Whitney *U* test.

**Results:**

A total of 9112 respondents (5411, 59.4%, from China and 3701, 40.6%, from the United States) who completed our survey were included in our analysis. After PSM, 1240 (22.9%) respondents from China and 1240 (33.5%) from the United States were matched for sex, age, educational level, occupation, and annual salary levels. The segmented sizes of 3 classes of respondents from China were 870 (70.2%), 270 (21.8%), and 100 (8.0%), respectively. Meanwhile, the US respondents’ segmented sizes were 269 (21.7%), 139 (11.2%), and 832 (67.1%), respectively. Respondents from China attached the greatest importance to the type of medical institution (weighted importance=40.0%), while those from the United States valued the waiting time (weighted importance=31.5%) the most. Respondents from China preferred the emergency department (coefficient=0.973, reference level: online consultation) and fever clinic (a special clinic for the treatment of fever patients for the prevention and control of acute infectious diseases in China; coefficient=0.974, reference level: online consultation), while those from the United States preferred private clinics (general practices; coefficient=0.543, reference level: online consultation). Additionally, shorter waiting times, COVID-19 nucleic acid testing arrangements, higher reimbursement rates, and lower costs were always preferred.

**Conclusions:**

Improvements in the availability of COVID-19 testing and medical professional skills and increased designated health care facilities may help boost potential health care seeking during COVID-19 and prevent unrecognized community spreading of SARS-CoV-2 in China and the United States. Moreover, to better prevent future waves of pandemics, identify undiagnosed patients, and encourage those undiagnosed to seek health care services to curb the pandemic, the hierarchical diagnosis and treatment system needs improvement in China, and the United States should focus on reducing diagnosis costs and raising the reimbursement rate of medical insurance.

## Introduction

COVID-19 was first reported in Wuhan, Hubei Province, China. It is caused by SARS-CoV-2 [1]. COVID-19, which had spread to more than 200 countries and regions as of May 2, 2022, was declared a public health emergency of international concern by the World Health Organization (WHO), with over 511 million confirmed cases and around 6 million confirmed deaths worldwide [2], having a devastating impact on the global economy, public health system, and health care services.

Patients with COVID-19 typically reported fever as the primary symptom, together with symptoms of upper respiratory tract infection, including cough, fatigue, and dyspnea, similar to the common cold and influenza [1,3,4]. Thus, researchers and clinicians faced numerous difficulties in quickly and accurately distinguishing COVID-19 from other respiratory infectious diseases in the early stages of the epidemic [5,6], especially when increasingly more infected individuals were asymptomatic [7,8]. So far, the COVID-19 nucleic acid test remains the gold standard for diagnosing COVID-19 and serves as the foundation for identifying, tracing, and isolating infected individuals [9]. With only enhanced surveillance and public health and social measures (PHSMs) to guard against COVID-19, a large proportion of those infected may still be undiagnosed and constantly spreading the virus in the community [10]. Therefore, it would be important to investigate the motivation of the public to undergo COVID-19 nucleic testing if infection were suspected.

China and the United States implemented different PHSMs during COVID-19. Compared with the United States, China enacted stricter actions, quickly locking down cities with confirmed community transmission, requiring face masks in public, and declaring national health insurance pay for all COVID-19–related costs [11-13]. Different types and levels of PHSMs may lead to differed attitudes toward, preferences for, and practices in the management of COVID-19 infection, leading to different transmission patterns of COVID-19 in the community. There is also an abundance of differences between the 2 countries in terms of medical structures (especially the health care system) and medical treatment, as well as others. These differences may lead to different preferences and variations among people in the 2 countries concerning medical treatment options, hence influencing their health-seeking behavior during COVID-19.

Factors have been identified that could influence the health care–seeking and utilization behavior of the patients [14,15]. On the one hand, the perception of disease severity and fear of infection, as well as the availability of appropriate health care facilities, lay the foundation of health-seeking behavior [16]. On the other hand, the delay in obtaining urgent health care may be due to personal experiences and anxieties over the COVID-19 pandemic, mandatory quarantines, national halt of mobility, mandatory lockdowns, and loss of income [17]. Moreover, the health-seeking behavior of those with fever during the pandemic may also be compromised by the potential stigma and discrimination [18]. The need to eliminate uncertainty motivates people to seek information and health care [19,20], and health care seeking can assist the patients better in making health decisions [21]. During COVID-19, a run on the medical resources was well noted in the world, and the failure to implement nucleic acid testing in the early stage led to widespread SARS-CoV-2 in the community and the late imposing of identification-tracing-isolation of those infected [22,23]. With medical resources directed to compensate for these newly emerging infectious diseases and health care facilities crowded with infectious patients, studies have identified the difficulties and burdens patients with diseases other than COVID-19 faced during the pandemic [24,25]. Nevertheless, the health-seeking behavior of those potentially infected with COVID-19 during the pandemic was not explored.

Therefore, given the political and cultural differences between China and the United States, as well as the 2 countries' disparate approaches to COVID-19 prevention and general medical insurance policies, our study aimed to investigate the preferences and choices of patients with fever for initial diagnosis in China and the United States during the COVID-19 pandemic using propensity score matching (PSM) and discrete choice experiment (DCE) analysis. This study focused on the availability of health care services that may influence the health care–seeking behavior of patients with fever during the pandemic, which may provide policymakers with insights to reform the health care system, better reallocate medical resources, and promote campaigns to encourage undiagnosed patients to undergo testing and may also provide practical guidance for preparing for any other future outbreaks.

## Methods

### Overview

This self-administered online cross-sectional study was conducted in China and the United States from January to March 2021. The questionnaire was constructed and administered using Lighthouse Studio version 9.8.1 (Sawtooth Software Inc). In the questionnaire, a total of 12 demographic questions and 7 DCE questions were included. First, demographic and socioeconomic information was collected, including age, sex, education level, annual income, and occupation, followed by 1 set of DCEs to investigate the respondents’ preferences for the initial diagnosis of fever during the COVID-19 pandemic using simulated scenarios of different diagnosis and treatment attributes. The questionnaire generally included 7 scenarios, with 1 fixed scenario and 6 hypothetical scenarios with fixed attributes and random levels, where the respondents were required to choose 1 option of 3 in each scenario.

First, the demographic idiosyncrasy of the 2 groups of respondents before and after PSM was presented. Later, the general preferences of the 2 groups of respondents were presented to show population-wide preferences for the initial diagnosis of fever during the pandemic; moreover, to compare the 2 groups of respondents from China and the United States, PSM was utilized to 1-to-1-match the respondents for 5 confounding variables (sex, age, income level, occupations, and educational level), aimed at comparing the preferences without being influenced by the confounding variables and demographic factors.

### Selection of Attributes and Levels

DCEs are now widely used in the fields of health care and public health [26,27]. The literature indicates that patients’ preferences strongly correlate with their willingness to use diagnosis, treatment services, and follow-up treatment [28]. By consulting several public health experts and reviewing the relevant literature [29-32], this study identified the following 6 attributes concerning diagnoses and treatment services, as well as their corresponding levels: (1) diagnosis and treatment medical institutions, (2) diagnosis and treatment personnel, (3) waiting time, (4) whether to test the nucleic acid of COVID-19 immediately, (5) medical expenses, and (6) reimbursement rate of medical expenses. The detailed attributes and their respective levels are presented in Table 1.

**Table 1:** Diagnosis attributes and their respective levels in this DCE^a^ (January-March 2021).

Diagnosis attribute	Description and levels
Types of clinics	Description: types of health care institutions that provide medical services during the COVID-19 pandemic Levels: telephone consultation, online consultation, emergency room, fever clinic, and private clinic (general practices)
Medical staff	Description: types of health care workers who can provide medical services, including diagnosis and treatment, for patients during the COVID-19 pandemic Levels: doctor, nurse, and paramedic
Waiting time	Description: time needed for a patient to receive medical consultation or other medical services to diagnose their fever during the COVID-19 pandemic Levels: 0, 15, 30, 45, 60, and 75 minutes
Immediate COVID-19 nucleic acid testing	Description: whether to undergo the SARS-CoV-2 nucleic acid test upon receiving medical services for fever during the COVID-19 pandemic Levels: Yes and no
Reimbursement ratio	Description: how much (%) of the medical expenses that patients spend on their medical services for fever could be reimbursed during the COVID-19 pandemic Levels: 0%, 20%, 40%, 60%, 80%, and 100%
Cost	Description: direct cost for medical services the patients receive for diagnosing and treating fever during the COVID-19 pandemic Levels: US $0, US $25, US $50, US $75, and US $100

^a^DCE: discrete choice experiment.

### Questionnaire and DCE Instrument Design

The questionnaire took 5-10 minutes to complete. Upon completing the questionnaire, each respondent immediately got a randomly generated 6-digit code without filling in any personal information. With this code, they received a preset US $0.10 on the Amazon Mechanical Turk (MTurk) platform as a reward. All respondents were required to be at least 18 years old and consented to participate by clicking the “agree to participate in the questionnaire” option before formally starting to answer the questionnaire. Before completing the questionnaire, all respondents were fully informed that this questionnaire was completely anonymous. Once the respondents agreed to take the questionnaire survey, they were informed that they voluntarily agreed to participate in the study and the questionnaire answers would be protected by privacy laws.

**Figure 1:**
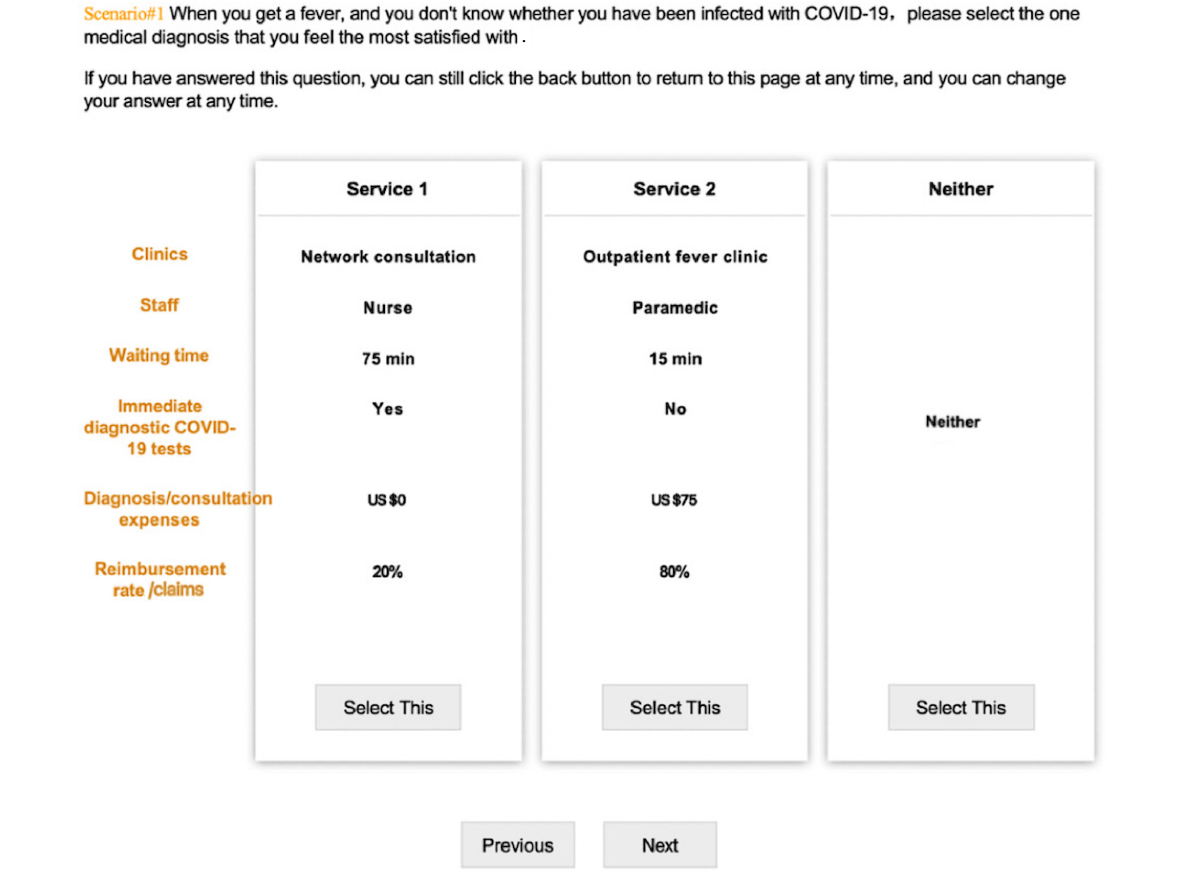
An example scenario of a choice-based conjoint in the questionnaire (January-March 2021).

In the first part of the questionnaire, respondents were required to provide basic demographic information, including sex, age, educational level, occupation, annual salary, and marital status. In addition to the basic demographic information, respondents were also asked whether they had ever been infected with COVID-19 and whether their acquaintances had ever been infected with COVID-19. The second part asked the respondents to consider a preferred treatment plan among 3 options in a task-choice scenario. Each scenario required the respondents to imagine themselves in a fever state and asked how they would seek health care services. This questionnaire included 6 attributes with a maximum of 6 levels, 7 scenarios per respondent, 3 alternatives per scenario. One example of the task-choice scenario is shown in Figure 1. See Multimedia Appendices 1 and 2 for the English and Chinese questionnaires, respectively.

Internal validity was evaluated using the program developed by Johnson et al [33], which includes stability (with repeated questions), within-set dominated pairs, across-set dominated pairs, transitivity, and attribute dominance (noncompensatory preferences). Multimedia Appendix 3, Table S9, shows information regarding the attributes of the DCE questionnaire, and Multimedia Appendix 1, Table S10, summarizes the test summaries. The internal validity test results and the summary results are shown in Multimedia Appendix 1, Tables S11-S13. According to the relevant research [34,35], our results showed that our questionnaire is efficient.

### Data Collection

Questionnaires were distributed via multiple international online panel providers (for data collection in the United States) and recruited volunteers (for data collection across China) from January to March 2021 [36,37]. Specifically, MTurk was used for data collection in the United States, and stratified sampling by age and geological locations was used for data collection in China [38]. MTurk was found to provide census-level sampling data during ongoing social events [39-41]. In total, 10,921 respondents participated in the survey, but only 9112 (83.4%) finished all the questions. Therefore, a total of 9112 respondents were included in the study, with 5411 (59.4%) respondents from China and 3701 (40.6%) from the United States. According to the rule of thumb [42], the sample size of a DCE depends on the number of choice tasks (t), the number of alternatives (a), and the number of analysis cells (c). According to the equation



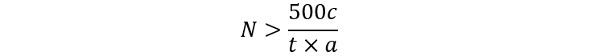



when considering the main effects, c equals the largest number of levels for any attribute. For this study, c=6, t=7, and a=3; thus, a minimum of 143 respondents were required.

### Statistical Analysis

Sociodemographic data were analyzed using descriptive statistics of frequency and percentage. Comparisons between the respondents from China and the United States were conducted using the chi-square test, and the results were presented as frequencies and percentages. Statistical significance was set at *P*<.05 (2-tailed). All the results were analyzed using STATA version 14.0 (Stata Corp), except the latent class models (LCMs) and assessment of internal validity of the DCE, which were analyzed using Lighthouse Studio version 9.12.1.

For the DCE, a mixed logit model (MXL) was first used to quantify the preferences of the respondents for the attributes and levels of an initial diagnosis of fever during COVID-19 in their trade-off in general. After using the MXL, we dummy-coded all the attribute levels, with the levels with the lowest model parameter *β* as the reference level in each attribute, by which we could enhance the interpretation of the preference weights by specifying the difference between 2 random coefficients.

PSM was performed to minimize the confounding bias of respondents from the United States and China that arose from the discrepancy of the demographic characteristics in both groups [38]. Specifically, the covariates were identified through the pairwise Pearson correlation matrix, and the final set of covariates for PSM was decided by minimizing the residual confounding factors as much as possible, where a logistic regression model was conducted to estimate the propensity scores for each group of respondents. Later, we conducted 1-to-1 matching without replacement so that a candidate respondent in the United States could be matched to only 1 respondent in China, after which the distribution of the covariates between the 2 groups would be the same [43]. Finally, a total of 2480 respondents, with 1240 (50%) from China and the other half from the United States, were matched from the total 9112 respondents, with the covariates being sex, age, occupation, educational level, and annual income. The flowchart of the PSM is shown in Multimedia Appendix 3, Figure S1.

The MXL was used to quantify the preference importance and weights of the various attributes of the DCE in the respondents' trade-offs. Additionally, the utility that the coefficients and SD used measured the levels of each attribute. The attribute cost was transformed into a continuous variable. Other parameters were assigned with a normal distribution, and we generated 1000 Halton draws for each population. We assumed that the attribute levels with *P*<.05 were statistically significant. We calculated each attribute preference's general estimated weight to identify its importance. The formula is:

Weighted importance = Coefficient of attribute X/Sum of coefficients of all attributes except the cost attribute

LCMs were used to explore the preference heterogeneity among the populations from the United States and China; this study also presented an LCM analysis, which divided the respondent population from the United States and China into a fixed proportion. Moreover, the number of latent groups was identified using the Akaike information criterion (AIC) and the Bayesian information criterion (BIC) [44]. In this study, 3 groups of the respondent population from China and 3 from the United States were identified and included in further research. In the study [45], we compared the models with 2-5 classes according to the AIC, the BIC, and the consistency information criterion (CAIC). Multimedia Appendix 3, Tables S3 and S4, show the AIC, BIC, and CAIC values of different classes in China and the United States.

The willingness to pay (WTP) is a measure used to capture the upper limit of the amount of money that people are willing to sacrifice to obtain the benefits of a particular medical service, diagnosis, and treatment plan—that is, the highest amount of money that respondents were willing to sacrifice when they chose their preferred diagnosis and treatment service in this study. Our study analyzed the WTP of the respondents to determine the homogeneity or heterogeneity caused by the cost in the choice of treatment options. We estimated the WTP:

WTPx = (vx_1_ – vx_0_)/−β_cost_,

where *β*_cost_ is the coefficient on the cost parameter and vx_0_ and vx_1_ are the coefficient before and after a change in the level of attribute x, respectively. For each reference attribute, vx_0_ was considered 0.

### Ethical Considerations

The respondents provided informed consent before filling in the questionnaire and agreed to participate in screening and to the use and publication of their data in journal papers. The questionnaire was completely anonymous, and the answers were protected by privacy law. During the process of filling in the questionnaire, all respondents could withdraw from the survey at any time. The study was conducted according to the guidelines of the 1964 Declaration of Helsinki and was approved by the Jinan University Medical Ethics Committee (JNUKY-2021-004). All procedures performed involving human respondents were in accordance with the ethical standards of the institutional and national research committee and with the 1964 Declaration of Helsinki and its later amendments or comparable ethical standards.

## Results

### Data Acquisition and Demographic Characteristics

A total of 9112 respondents from China and the United States were included in the final analysis, the demographic characteristics of whom are shown in Table 2. Of these respondents, 5411 (59.4%) respondents were from China and 3701 (40.6%) respondents were from the United States. After PSM, 1240 (22.9%) respondents from China and 1240 (33.5%) from the United States were matched, and no apparent differences were found between the 2 groups of respondents (*P*>.05 for all sociodemographic factors), as shown in Table 2.

After PSM, of the 1240 respondents from China, 1188 (95.8%) were between 18 and 60 years old and 706 (56.9%) were female. Of those from the United States, 1182 (95.3%) were between 18 and 60 years old, 705 (56.9%) were female, and 18 (1.5%) had a postgraduate degree.

**Table 2:** Demographic characteristics of nonmatched and propensity score–matched respondents from China and the United States (January-March 2021).

Baseline matching characteristics		Nonmatched respondents					Propensity score–matched respondents	
		China (n=5411), n (%)		United States (n=3701), n (%)		China (n=1240), n (%)		United States (n=1240), n (%)
**Sex (nonmatched *P*=.003; propensity score–matched *P*=.99)**								
	Male	2400 (44.4)	1765 (47.7)		534 (43.1)			535 (43.1)
	Female	2993 (55.3)	1918 (51.8)		706 (56.9)			705 (56.9)
	Other	18 (0.3)	18 (0.5)		0			0
**Age (nonmatched *P*<.001; propensity score–matched *P*=.99)**								
	18-25	1127 (20.8)	501 (13.5)		164 (13.2)			162 (13.1)
	26-30	762 (14.1)	762 (20.6)		235 (19.0)			234 (18.9)
	31-35	704 (13.0)	750 (20.3)		244 (19.7)			251 (20.2)
	36-40	490 (9.1)	505 (13.6)		152 (12.3)			152 (12.3)
	41-45	520 (9.6)	368 (9.9)		139 (11.2)			136 (10.9)
	46-50	632 (11.7)	241 (6.5)		105 (8.5)			103 (8.3)
	51-55	434 (8.0)	174 (4.7)		89 (7.2)			84 (6.8)
	56-60	349 (6.4)	154 (4.2)		60 (4.8)			60 (4.8)
	>60	393 (7.3)	246 (6.7)		52 (4.2)			58 (4.7)
**Highest educational level (nonmatched *P*<.001; propensity score–matched *P*=.87)**								
	Preprimary education or primary school education	404 (7.5)	2 (0.1)		1221 (98.5; nonpostgraduate)			1222 (98.5; nonpostgraduate)
	Middle school education	596 (11.0)	15 (0.4)		N/A^a^			N/A
	High school education	939 (17.4)	675 (18.2)		N/A			N/A
	Vocational school education	896 (16.6)	508 (13.7)		N/A			N/A
	Bachelor’s degree	2027 (37.5)	1710 (46.2)		N/A			N/A
	Master’s degree	428 (7.9)	711 (19.2)		N/A			N/A
	PhD	121 (2.2)	80 (2.2)		19 (1.5; postgraduate)			18 (1.5; postgraduate)
**Occupation and working area (nonmatched *P*<.001; propensity score–matched *P*=.99)**								
	Students	1238 (22.9)	249 (6.7)		139 (11.2)			132 (10.6)
	Managers	685 (12.7)	5419 (14.6)		178 (14.4)			174 (14.0)
	Professionals	775 (14.3)	93 (2.5)		250 (20.2)			250 (20.2)
	Technicians and associate professionals	798 (14.8)	423 (11.4)		148 (11.9)			157 (12.7)
	Clerical support workers	232 (4.3)	318 (8.6)		121 (9.8)			122 (9.8)
	Service and sales workers	521 (9.6)	453 (12.2)		185 (14.9)			188 (15.2)
	Skilled agricultural, forestry, and fishery workers	378 (7.0)	43 (1.2)		14 (1.1)			14 (1.1)
	Craft and related trade workers	122 (2.3)	78 (2.1)		27 (2.2)			28 (2.3)
	Plant and machine operators and assemblers	184 (3.4)	32 (0.9)		11 (0.9)			11 (0.9)
	Elementary occupations	133 (2.5)	75 (2.0)		16 (1.3)			14 (1.1)
	Armed forces occupations	73 (1.4)	19 (0.5)		6 (0.5)			4 (0.3)
	Other	272 (5.0)	477 (12.9)		145 (11.7)			146 (11.8)
**Annual salary level (US $; nonmatched *P*<.001; propensity score–matched *P*=.99)**								
	**<**10,000	2272 (48.1)	398 (11.0)		335 (27.0)			333 (26.9)
	10,001-20,000	1232 (26.1)	382 (10.6)		257 (20.7)			259 (20.9)
	20,001-30,000	564 (11.9)	481 (13.3)		236 (19.0)			236 (19.0)
	30,001-40,000	297 (6.3)	472 (13.1)		192 (15.5)			193 (15.6)
	40,001-50,000	164 (3.5)	456 (12.6)		91 (7.3)			92 (7.4)
	50,001-60,000	55 (1.7)	464 (12.8)		41 (3.3)			40 (3.2)
	60,001-70,000	47 (1.0)	331 (9.2)		23 (1.9)			24 (1.9)
	>70,000	94 (2.0)	630 (17.4)		65 (5.2)			63 (5.1)

^a^N/A: not applicable.

### General MXL Results

**Figure 2:**
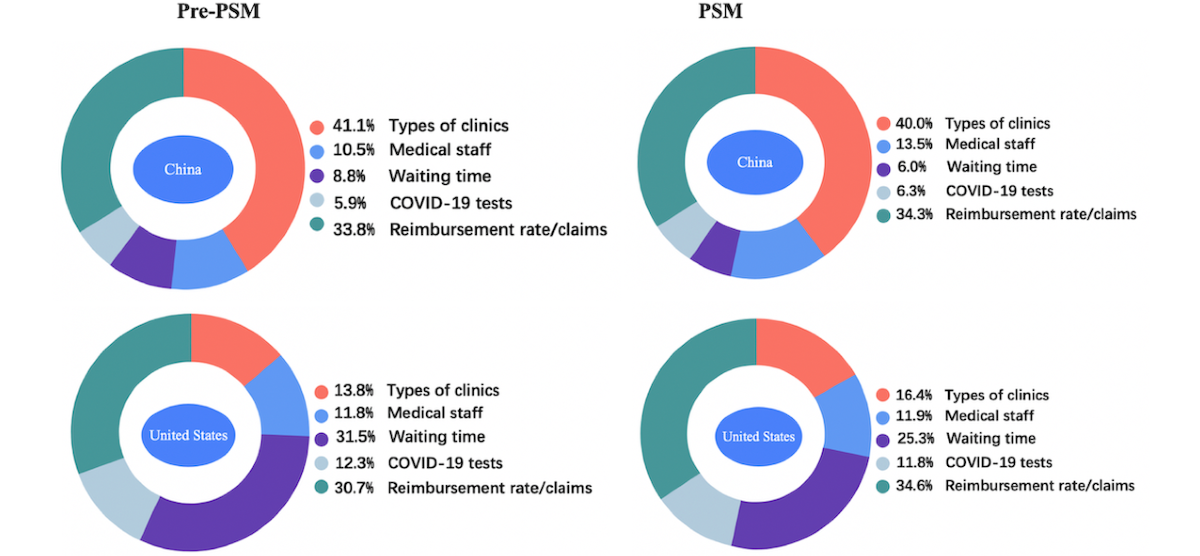
General estimated weighted importance of attribute preference in pre-PSM and PSM respondents in China and the United States (January-March 2021). PSM: propensity score matching.

The comparison of relative attribute importance between China and the United States before and after PSM is shown in Figure 2. After PSM, respondents from China attached the most importance to the types of the medical institutions (39.9%), followed by the reimbursement rate (34.3%), and the waiting time was the least essential attribute (6.5%). For respondents from the United States, the reimbursement rate was the most important attribute (34.6%), followed by the waiting time (25.3%).

The MXL results depicting the levels of each attribute of respondents' preferences in China and the United States for an initial diagnosis of fever during the COVID-19 pandemic before and after PSM are shown in Tables 3 and 4, respectively. Respondents from China strongly preferred going to a fever clinic (utility coefficient=0.974) or the emergency department (utility coefficient=0.973) compared to a network consultation. In contrast, US respondents preferred private clinics (general practices) the most. The more negative correlation of cost for the Chinese respondents showed that they cared more about the cost than the US respondents did. In addition, both populations showed a similar preference for immediate COVID-19 nucleic acid tests with a high reimbursement rate, which indicates that people consistently prefer low-consumption treatment plans.

**Table 3:** Pre-PSM^a^ results of the MLX^b^ model of the preferences of respondents in China (N=5411) and the United States (N=3701) for initial diagnosis of fever during COVID-19 (January-March 2021).

Attributes and levels		China					The United States					
		Coefficient	SD	SE	*P* value	Coefficient		SD	SE		*P* value	
**Mean**												
	Opt out (respondents chose neither of the two options)	–2.690	4.361	0.122	<.001	–2.344		4.134	0.136		<.001	
**Types of clinics**												
	Online consultation	Reference	N/A^c^	N/A	N/A	Reference		N/A	N/A		N/A	
	Private clinic	0.028	1.008	0.040	.48	0.471		0.679	0.047		<.001	
	Telephone consultation	0.292	0.364	0.036	<.001	0.030		0.948	0.048		.54	
	Fever clinic	1.124	1.103	0.045	<.001	0.322		0.298	0.044		<.001	
	Emergency room	1.011	0.804	0.043	<.001	0.050		0.698	0.046		.28	
**Medical staff**												
	Paramedic	Reference	N/A	N/A	N/A	Reference		N/A	N/A		N/A	
	Nurse	0.127	0.245	0.027	<.001	0.209		0.209	0.033		<.001	
	Doctor	0.499	0.785	0.030	<.001	0.533		0.732	0.038		<.001	
**Waiting time (minutes)**												
	75	Reference	N/A	N/A	N/A	Reference		N/A	N/A		N/A	
	0	0.172	0.509	0.041	<.001	0.631		0.635	0.053		<.001	
	15	0.175	0.388	0.041	<.001	0.530		0.395	0.051		<.001	
	30	0.093	0.477	0.041	.02	0.431		0.415	0.051		<.001	
	45	0.055	0.280	0.041	.18	0.260		0.305	0.051		<.001	
	60	0.030	0.182	0.040	.46	0.140		0.349	0.051		.006	
**COVID-19 nucleic acid testing**												
	No	Reference	N/A	N/A	N/A	Reference		N/A	N/A		N/A	
	Yes	0.350	0.899	0.024	<.001	0.774		1.111	0.037		<.001	
**Reimbursement ratio (%)**												
	0	Reference	N/A	N/A	N/A	Reference		N/A	N/A		N/A	
	20	0.130	0.377	0.041	.002	0.029		0.431	0.051		.56	
	40	0.187	0.338	0.041	<.001	0.176		0.194	0.049		<.001	
	60	0.340	0.054	0.041	<.001	0.346		0.137	0.050		<.001	
	80	0.584	0.523	0.042	<.001	0.560		0.591	0.052		<.001	
	100	0.776	0.928	0.045	<.001	0.825		1.119	0.057		<.001	
Cost		–6.53	2.05	0.190	<.001	–5.74		1.78	0.144		<.001	

^a^PSM: propensity score matching.

^b^MXL: mixed logit model.

^c^N/A: not applicable.

**Table 4:** Post-PSM^a^ results of the MLX^b^ model of the preferences of respondents in China (N=1240) and the United States (N=1240) for initial diagnosis of fever during COVID-19 (January-March 2021).

Attributes and levels			China								The United States						
			Coefficient	SD		SE		*P* value		Coefficient			SD		SE		*P* value
**Mean**																	
	Opt out (respondents chose neither of the two options)	–2.663		4.066	0.245		<.001		–2.045			4.550		0.239		<.001	
**Types of clinics**																	
	Online consultation	Reference		N/A^c^	N/A		N/A		Reference			N/A		N/A		N/A	
	Private clinic	0.054		1.054	0.084		.52		0.543			0.854		0.091		<.001	
	Telephone consultation	0.208		0.181	0.076		.01		0.070			1.131		0.093		.45	
	Fever clinic	0.974		1.140	0.096		<.001		0.434			0.581		0.087		<.001	
	Emergency room	0.973		0.757	0.091		<.001		0.069			0.931		0.090		.44	
**Medical staff**																	
	Paramedic	Reference		N/A	N/A		N/A		Reference			N/A		N/A		N/A	
	Nurse	0.136		0.151	0.056		.02		0.237			0.496		0.064		<.001	
	Doctor	0.609		0.981	0.070		<.001		0.570			0.838		0.074		<.001	
**Waiting time (minutes)**																	
	75	Reference		N/A	N/A		N/A		Reference			N/A		N/A		N/A	
	0	0.144		0.566	0.087		.10		0.136			0.875		0.095		.16	
	15	0.154		0.643	0.088		.08		0.136			0.535		0.099		.17	
	30	0.032		0.254	0.085		.71		0.397			0.176		0.097		<.001	
	45	-0.017		0.439	0.087		.85		0.401			0.665		0.097		<.001	
	60	0.020		0.357	0.086		.82		0.649			0.063		0.103		<.001	
**COVID-19 nucleic acid testing**																	
	No	Reference		N/A	N/A		N/A		Reference			N/A		N/A		N/A	
	Yes	0.348		0.902	0.051		<.001		0.801			1.126		0.069		<.001	
**Reimbursement ratio (%)**																	
	0	Reference		N/A	N/A		N/A		Reference			N/A		N/A		N/A	
	20	0.103		0.540	0.089		.25		0.107			0.843		0.100		.28	
	40	0.198		0.429	0.087		.02		0.310			0.364		0.096		.001	
	60	0.286		0.217	0.085		.001		0.449			0.146		0.096		<.001	
	80	0.566		0.513	0.089		<.001		0.549			0.885		0.100		<.001	
	100	0.742		1.037	0.098		<.001		0.935			0.994		0.108		<.001	
Cost			–6.81	1.98		0.37		<.001		–5.52			1.86		0.196		<.001

^a^PSM: propensity score matching.

^b^MXL: mixed logit model.

^c^N/A: not applicable.

### Willingness-to-Pay Results

A WTP greater than 0 indicates that the WTP can ensure a change in the reference level, while a WTP less than 0 indicates the patients are willing to pay to avoid a change in the reference level. Through the analysis, it was evident that the Chinese respondents preferred hospital emergency and fever clinics, for which they were willing to pay US $0.14 (reference level: online consultation US $0) and US $0.14 (reference level: online consultation US $0) to receive services from these 2 types of medical institutions. At the same time, they are willing to pay US $0.09 (reference level: paramedic US $0) for the treatment provided by doctors. Compared to the WTP of the respondents from China, the respondents from the United States were more willing to pay US $0.10 (reference level: online consultation US $0) and US $0.08 (reference level: online consultation US $0) for treatment in private hospitals and fever clinics, revealing a preference discrepancy with China. Both US and Chinese respondents were willing to pay US $0.15 and US $0.05 for immediate COVID-19 nucleic acid testing (Tables 5 and 6). They were willing to pay a certain amount for a shorter waiting time and a higher reimbursement rate, indicating that a diagnosis service with an immediate COVID-19 nucleic acid test, shorter waiting time, and lower cost is more acceptable for respondents. Specifically, the interaction test indicated that cost and reimbursement rate have a significant interaction. This indicates that the effects of choice obtained with the 2 attributes vary together (Multimedia Appendix 3, Tables S5-S8).

**Table 5:** Respondents’ WTP^a^ in China (January-March 2021).

Attribute and change		WTP (US $)
**Types of clinics**		
	Online consultation–private clinic	0.01
	Online consultation–telephone consultation	0.03
	Online consultation–fever clinic	0.14
	Online consultation–the emergency room	0.14
**Medical staff**		
	Paramedic-nurse	0.02
	Paramedic-doctor	0.09
**Waiting time (minutes)**		
	75-0	0.02
	75-15	0.02
	75-30	0.00
	75-45	0.00
	75-60	0.00
**COVID-19 nucleic acid testing**		
	No-yes	0.05
**Reimbursement ratio (%)**		
	0-20	0.02
	0-40	0.03
	0-60	0.04
	0-80	0.08
	0-100	0.11

^a^WTP: willingness to pay.

**Table 6:** Respondents’ WTP^a^ in the United States (January-March 2021).

Attribute and change		WTP (US $)
**Types of clinics**		
	Online consultation–private clinic	0.10
	Online consultation–telephone consultation	0.01
	Online consultation–fever clinic	0.08
	Online consultation–the emergency room	0.01
**Medical staff**		
	Paramedic-nurse	0.04
	Paramedic-doctor	0.10
**Waiting time (minutes)**		
	75-0	0.02
	75-15	0.02
	75-30	0.07
	75-45	0.07
	75-60	0.12
**COVID-19 nucleic acid testing**		
	No-yes	0.15
**Reimbursement ratio (%)**		
	0-20	0.02
	0-40	0.06
	0-60	0.08
	0-80	0.10
	0-100	0.17

^a^WTP: willingness to pay.

### LCM Results

After comparing the AIC and BIC, we determined 3 classes for respondents from China and 3 for those from the United States. The segmented sizes of the 3 classes of respondents from China were 870 (70.2%), 270 (21.8%), and 100 (8.0%), respectively. The US respondents’ segmented sizes were 269 (21.7%), 139 (11.2%), and 832 (67.1%), respectively.

**Figure 3:**
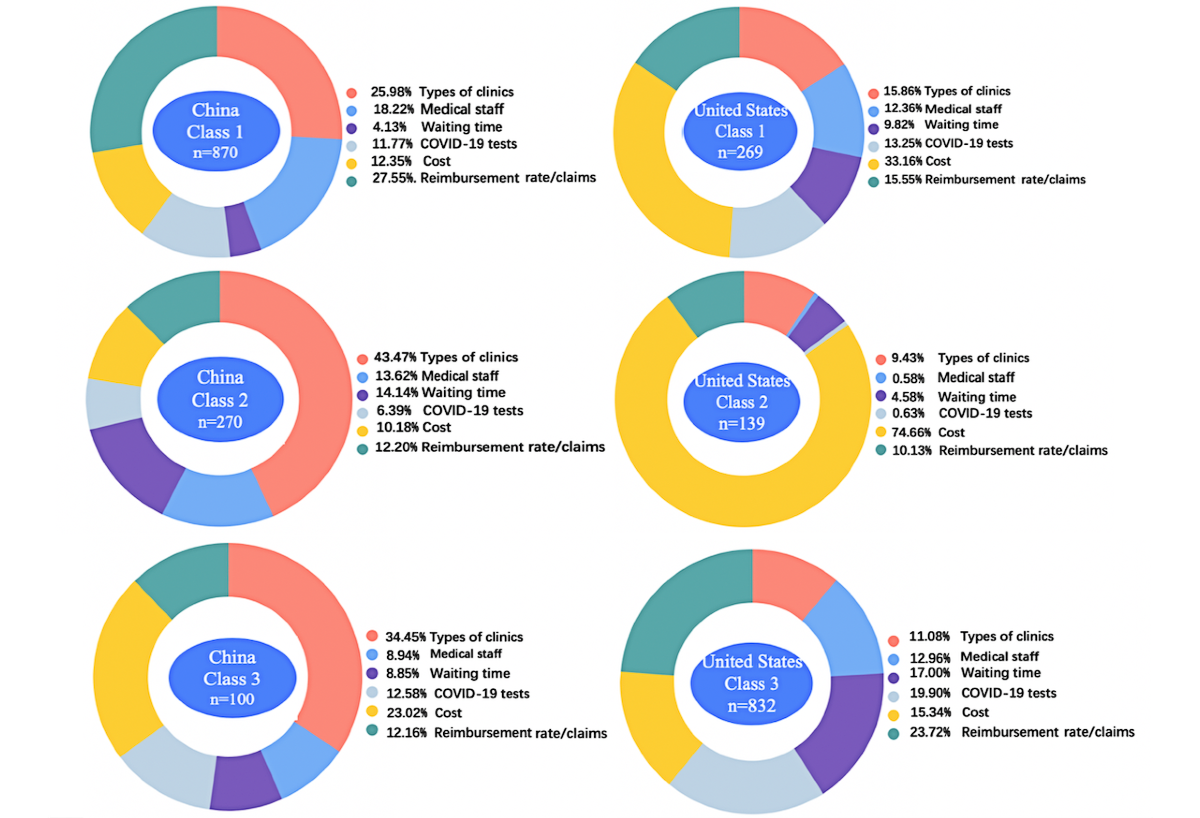
Weighted importance of diagnosis attributes in China and the United States, as determined by the LCM (January-March 2021). LCM: latent class model.

**Figure 4:**
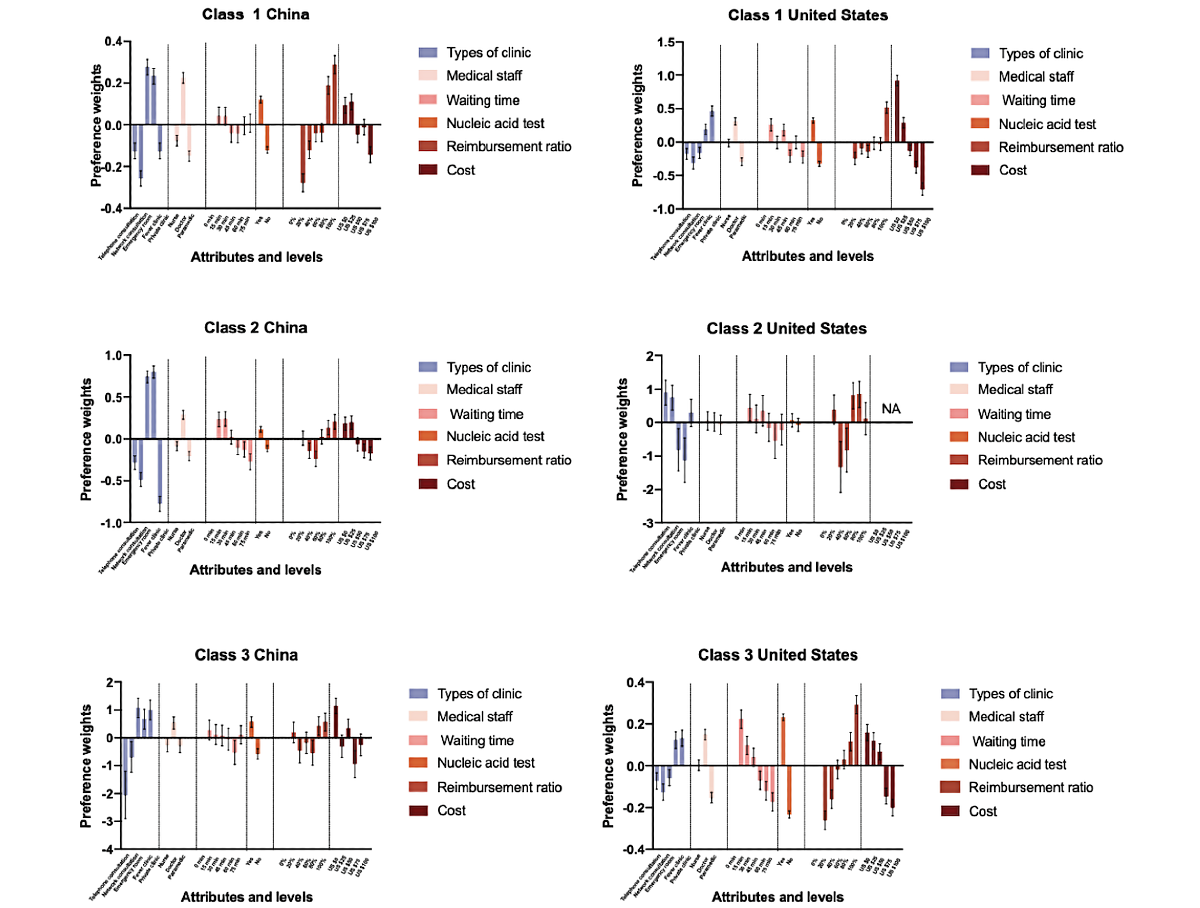
Preference weights stratified by group and class in China and the United States (January-March 2021).

Figure 3 shows the heterogeneities of attribute importance of different classes of respondents from China and the United States, and Figure 4 shows preference weights stratified by group and class. Class 1 of respondents from China ranked reimbursement rate and claims as the first important attribute, while classes 2 and 3 thought that the importance of the types of clinics is the most critical factor affecting their medical preference. Meanwhile, classes 1 and 3 of respondents from China considered the waiting time the least important, while class 2 of respondents from China ranked the immediate COVID-19 test as the least important attribute.

For the classes of respondents from the United States, classes 1 and 2 ranked cost as the first important attribute, while class 3 attached the most importance to the reimbursement rate and claims. For these 3 classes of respondents from the United States, the least important attributes were waiting time, types of staff, and types of clinics, respectively. Cost had overwhelming importance in class 2 compared to the other 2 classes.

## Discussion

### Principal Findings

The study found that respondents from China and the United States had distinctly different preferences for attributes regarding the initial diagnosis of fever during the COVID-19 pandemic. Types of medical institutions were the most important factor for Chinese respondents, while those from the United States thought that cost was the most important factor when seeking medical services for fever during the pandemic. In addition, both populations highlighted the importance of the reimbursement rate. These heterogeneities and homogeneities may result from differences in the medical systems, health care services provided, COVID-19–mitigating strategies, and medical insurance systems between China and the United States.

### Comparison With Prior Work

DCEs can be used to understand people's underlying psychological situations, and the influencing factors and weights of choice preferences can be obtained through the hypothetical medical choice [46]. DCEs are also widely used in a series of aspects such as epidemic prevention, control, and supervision [47-49]. To the best of our knowledge, this is the first study to explore the preferences for health care services for the initial diagnosis of patients with fever during the COVID-19 pandemic. Our previous work found that respondents in China had a rather considerable basic knowledge of the detection methods of SARS-CoV-2 and the types of testing kits, even if they have no experience in contracting the virus or undergoing screening tests [50]. Nevertheless, considering that during the pandemic, everyone in China seeking health care services for fever, together with those accompanying them, would be screened by the SARS-CoV-2 nucleic acid test [51], the Chinese respondents placed the least importance on the necessity of immediate nucleic acid tests, while for the US respondents, COVID-19 tests accounted for 12.3% of the relative importance.

According to Caldow et al [52], patients prefer medical services provided by doctors, which is consistent with our results that doctors are preferred over nurses and paramedics for diagnosing fever [52]. During the pandemic, to relieve the consultation pressure of fever clinics in hospitals and prevent cross-infection, online fever clinics, an internet-based clinic system, were utilized in China, where a study enrolling more than 60,000 patients found that online fever clinics may efficiently ease patients' worry and clinicians can educate patients who are suspected of having COVID-19 to isolate and protect themselves [53]. Online and telephone consultation services have the characteristics of convenience and rapidity and can transcend distances to achieve preliminary medical services. In the case of future outbreaks, public health guidelines and policymaking may incorporate these 2 services into the first step of medical services to quickly divert different patients to curb the population contact transmission of infectious diseases. Although the respondents did not readily choose and prefer online clinics, Zhao et al [54] found that during the pandemic, many patients had trouble obtaining offline health care services and relied heavily on the internet for health information [54].

### China and the United States Hold Distinctly Different Organization and Governance of the Health System

Chinese respondents regarded the medical institution type as the most important preference factor, especially public medical institutions, while respondents from the United States preferred private medical institutions, as shown in Table 4. The phenomenon may be attributable to the different medical and health service systems of China and the United States [55].

In China, health care providers include hospitals, primary health care institutions, and specialized public health institutions, where government-owned public hospitals and health institutions remain dominant in providing health care services, accounting for around 60% of all hospitals [56]. Nevertheless, the US health care system is more a combination of multiple systems operating individually but synergically, where nongovernment entities play a vital role in building the industry standard, fostering health accessibility, improving the quality of life, and controlling costs at various levels. With more than 6000 hospitals registered in the United States, only about 200 hospitals are owned by the federal government. The majority (more than 5000) of the hospitals are community hospitals, which include nonfederal hospitals, short-term general hospitals, and other special hospitals [57].

In this sense, it would be reasonable to assume that Chinese citizens would choose government-owned health institutions, while US citizens would prefer private-owned hospitals, which corresponds to the notion that government-owned hospitals predominate in China and community hospitals prevail in the United States. This fact consistently correlates with our results in Table 3, which show that Chinese respondents preferred fever clinics (designated public hospitals specializing in managing feverish patients during the pandemic), while the US respondents tended to choose private clinics.

According to LCM results, all respondents (from both China and the United States) were more willing to accept the diagnosis and treatment scheme with immediate nucleic acid testing, lower treatment costs, and higher reimbursement rates. In the LCM results, class 3 in China showed a preference for private medical institutions, and class 2 in the United States was more willing to receive telephone consultations than to travel to medical institutions; these findings differed from the overall performances of the Chinese and US groups.

Since the health system reform was enacted in 2009, more private-owned hospitals and health institutions have been established to provide the general population with equal access to basic health care [58-60]. According to a report by the China Statistical Information Center, from January to February 2021, the outpatient volume in China reached 960 million people, including 510 million in public hospitals and 90 million in private hospitals [61].

Despite its uneven distribution of medical resources in urban and rural areas, China is gradually beginning to promote internet hospitals. Telephone and online consultations have grown rapidly during the COVID-19 pandemic. Internet hospitals enable patients to consult doctors from large university-affiliated public hospitals for treatment through other internet hospitals [62]. During the pandemic, the application of internet hospitals in China, together with a remote drug delivery platform, has helped maintain constant health care services and provide for those in need, specifically those with chronic and mild diseases. This has been considered a potent tool to optimize medical resource distribution by relieving offline hospitals and catering to those in need without contracting the virus [63].

However, telephone and online consultations are still in their early stage of development. Because of the lack of standardized diagnosis and treatment standards, poor operation, and management issues, these 2 consultation schemes cannot wholly replace the conventional diagnostic process. Compared to traditional face-to-face medical schemes, the number of patients they serve is still small [64-66]. Nevertheless, the low selection rate of these 2 consultation modes may be attributable to the respondents having no idea of how these 2 types of consultation work and how they provide health care services [67].

COVID-19 is inherently not a disease that can solely be diagnosed via symptoms and signs, and the internet hospital is still in its exploration stage, where various issues remain unsolved. Thus, not being preferred does not necessarily mean that the internet hospital is suboptimal in guiding patients toward effective medical treatment.

### Distinctly Different Financing Methods for Health Care Services in China and the United States

Additionally, for respondents from the United States, cost was the most important factor affecting preference. This may be caused by the differences in the treatment costs and medical reimbursement systems in China and the United States.

In the United States, only around 30% of the population is covered by the public financing system, mainly via Medicare and Medicaid, and around 54% of the population receives private health insurance [68]. Nevertheless, one-sixth of Americans are uninsured, and high out-of-pocket expenditure still may put a heavy burden on some of those receiving insurance, hindering timely health care and medications.

Nevertheless, in China, the Information Office of the State Council of the People's Republic of China announced that the cost of COVID-19 in China would be covered by the national free treatment policy [69,70]. Therefore, it is reasonable that the respondents from China do not attach the maximum importance to the cost of diagnosis and treatment is the most important factor. This may be due to the national free treatment policy, which helps eliminate the burden of treatment costs on the public.

COVID-19 patients with severe and nonsevere complications were admitted to hospitals at an average cost of US $20,292 according to data provided by the Kaiser Family Foundation in the United States, and this is about 8.5 times the average cost in China. At the same time, although there is a medical insurance system in the United States, even after Medicare reimbursement, the average out-of-pocket expenses of patients may exceed US $1300 [71]. Experts have recommended that new federal legislation should be established to expend federal funds on emergency responses, hiring and training of personnel, and distribution of diagnostic tests, therapeutic approaches, and vaccines at different levels and, most importantly, to expand the coverage rate of medical insurance for diagnosing, treating, and following up patients with COVID-19 [72]. During COVID-19, a wide range of previously unavailable telehealth services were covered by Medicare and Medicaid, allowed by the Section 1135 waiver in the United States [73]. Moreover, the havoc COVID-19 caused on the economy resulted in a 15% unemployment rate in May 2020 [74], which increased the rate of the uninsured and enrollment in Medicaid, and hence some have addressed the need to put forward “Medicare for All” [75]. Nevertheless, scholars have addressed that ensuring effective government action with sensible private sector regulation may be a preferable option over turning to insurance to pay for COVID-19–related medical costs [76]. Generally, we believe that both expanding the coverage of insurance to those uninsured or with low insurance for COVID-19–related health care costs and ensuring the federal mandate for free access to COVID-19 testing and treatment can effectively motivate those potentially infected to undergo testing and proper treatments.

### Encouraging Undiagnosed Patients to Test for COVID-19 is Important for Curbing the Pandemic

Identification, tracing, and isolation of those infected are vital for containing the community spread of COVID-19. Nevertheless, if those infected remain untested and no universal nucleic test programs are conducted, then the infected citizens may still be constantly spreading the virus in the community. Therefore, identifying potential COVID-19–infected patients in the community and encouraging them to undergo testing and quarantine is important for curbing COVID-19 spread in the community.

In China, large-scale community nucleic acid testing can be implemented to identify potentially infected people due to the state's attitude toward early diagnosis, early isolation, and early treatment of COVID-19. The willingness of the public to participate in screening tests depends on their awareness of the risks and benefits. The governmental entities stipulate that residents in controlled areas must participate in community nucleic acid testing and those who do not participate without good cause may suffer legal consequences and difficulties in daily life [51]. This is in line with the general policy of “dynamic zero COVID-19 strategies” for preventing and controlling the former COVID-19 pandemic in China [77].

For the United States and other noncentralized countries where large-scale community-wide mass screening tests are difficult to implement, it is difficult to identify those infected in the community unless they undergo screening tests voluntarily. However, some people still avoid nucleic acid testing as they are afraid of testing positive for the disease and other factors [78]. Although the Centers for Disease Control and Prevention (CDC) and other institutions have issued a series of guidelines on coping with COVID-19 [79], according to Park et al [80], the psychological pressure of contracting COVID-19 may reduce the rate of public compliance with official health guidelines. Therefore, it is recommended that the public be encouraged to participate in screening tests, either nucleic acid tests or antigen tests, in a number of ways, including broadcasting public messages by medical and health experts, providing information on social media, and distributing small gifts to those who participate in testing, to make every resident aware of the importance and obligation of nucleic acid screening [81,82]. Improving the participation rate in those screening, testing, and detecting of those infected may help control the spread of COVID-19. We advocate the use of publicity campaigns in the media, the spread of rigorous scientific information, the promotion of culturally sensitive psychological counseling, and other related services to account for different needs and to encourage the public to be willing to participate in screening tests. Regarding the stigma and discrimination related to being diagnosed as COVID-19 positive, it is necessary to provide social support to relieve the potential stigma and social unrest. To make this possible, multidisciplinary teams comprising experts from clinics, social sciences, government entities, communication, and the media are needed [83].

### Limitations

There are limitations of our research. First, the nature of this cross-sectional study inherently led to reporting bias, information bias, and confounding bias. In the study, we used a closed-end, self-administered questionnaire to prevent missed data and used online an panel platform (MTurk) to prevent selection bias, as a previous study proved the census-level quality of survey data collected via MTurk [61,84]. Nevertheless, selection bias may still exist. PSM was used to control the confounding effects when directly comparing the 2 cohorts of respondents. However, as various factors may underlie the respondents' decision-making in the 2 distinctly different countries, the scale differences may not be completely accounted for, and the results should be interpreted with caution. Moreover, as the DCE asked participants to make choices between hypothetical scenarios, which may not reflect real-world situations and hypothetical bias may exist, and we did not investigate external validity in the study, the results of the study should be interpreted cautiously. Our questionnaire involves the treatment modes of telephone and online consultations, which some respondents may not have experienced and may have led to selection bias. Moreover, we did not include questions on past experience with online/telephone consultation as well as previous experience with COVID-19 testing or treatment, so how such underlying factors may influence preferences could not be distinguished and need future exploration. In addition, we did not include the investigation of how different ethnicities and residence locations (urban and rural) may affect the respondents' preferences, which can be further explored in a future study. The significant interactions between cost and reimbursement rate render interpreting these 2 attributes difficult, so the WTP should be interpreted cautiously.

### Conclusion

Improvements in the availability of COVID-19 testing, medical professional skills, and designated health care facilities may help boost potential health care seeking during COVID-19 and prevent unrecognized community spread of SARS-CoV-2 in China and the United States. Moreover, to better prevent future waves of pandemics, identify undiagnosed patients, and encourage them to seek health care services to curb the pandemic, it is suggested that the hierarchical diagnosis and treatment system be improved in China and that the United States focus on reducing medical costs and raising the reimbursement rate of medical insurance. Second, online and telephone consultations may serve as patients' primary medical services, which may triage suspected and nonsuspected patients of infectious diseases, reducing the possible cross-infection during the pandemic.

## Appendix

Multimedia Appendix 1 DCE questionnaires in English.

Multimedia Appendix 2 DCE questionnaires in Chinese.

Multimedia Appendix 3 The new version of appendix for the manuscript .

## Abbreviations

AIC: Akaike information criterion
BIC: Bayesian information criterion
CAIC: consistency information criterion
CDC: Centers for Disease Control and Prevention
DCE: discrete choice experiment
LCM: latent class model
MTurk: Mechanical Turk
MXL: mixed logit model
PHSM: public health and social measure
PSM: propensity score matching
WTP: willingness to pay
