# Supplementary material for: Preferences for Attributes of Initial COVID-19 Diagnosis in the United States and China During the Pandemic: Discrete Choice Experiment With Propensity Score Matching
Source: JMIR Public Health Surveill. 2022 Aug 16;8(8):e37422. doi: 10.2196/37422 (PMC9384860; doi:10.2196/37422)
Supplement: Multimedia Appendix 1 [file publichealth_v8i8e37422_app1.pdf]

Start

Title

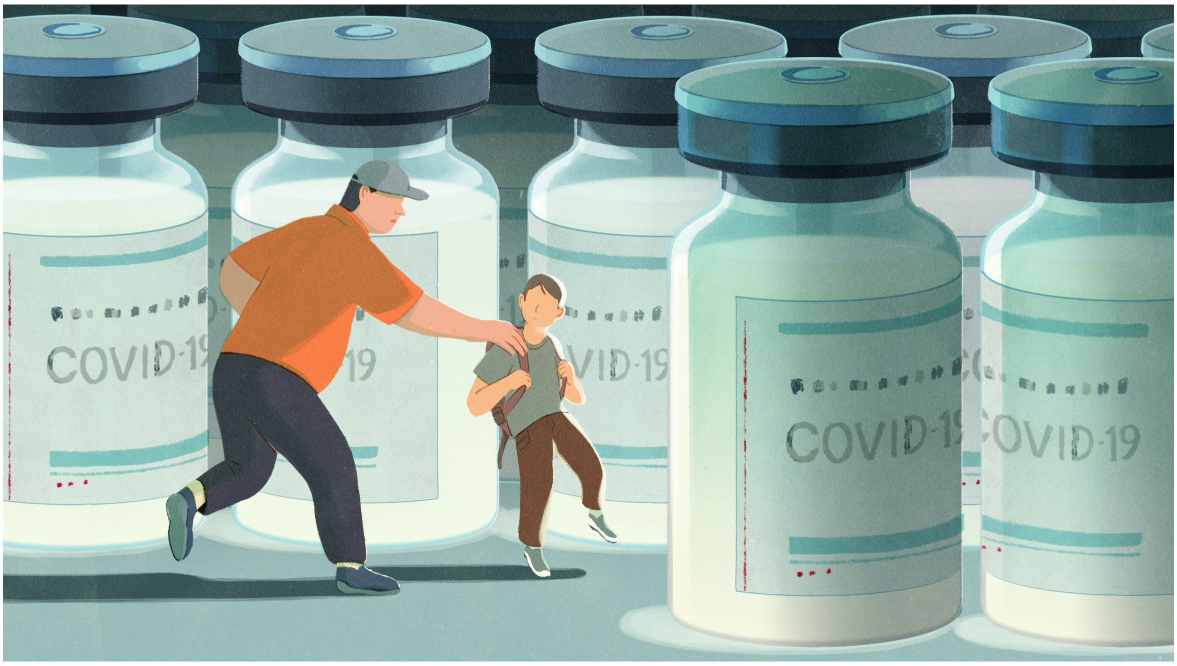

COVID-19 vaccination willingness and acceptance

Intro

Background

The COVID-19 pandemic has caused profound effects on the societies and economies globally. Scientists and pharmaceutical companies have been working on vaccines for COVID-19. Reports on several vaccines have been released and some countries have initiated vaccination programmes.

This survey aims to investigate acceptance and willingness to accept the COVID-19 vaccination among the public.

Next

Preface

Thank you for your participation! This questionnaire consists of 3 sections of questions and can be completed in about 10 minutes. You can exit this questionnaire at any time. We will keep your information confidential.

Previous Next

0% 100%

Consent

Hello,

you are about to participate in our questionnaire. This questionnaire will include several parts and will only take no more than 10 minutes to complete. Do you consent to undergo the questionnaire?

- Consent=1 ○ Agree
- Consent=2 ○ Disagree

AgeConsent

Are you above 18 years old?

- AgeConsent=1 ○ Yes
- AgeConsent=2 ○ No

This research does not include any funds from biomedical companies. If you have any questions, please feel free to send an email to t.liu.10@student.rug.nl.

COVID-19 Vaccinaton willingness research team

Previous | Next

0% 100%

Q1

Section 1. Demographic information

QS11

1. What is your gender?

QS11=1 ◯ Male

QS11=2 ◯ Female

QS11=3 ◯ Other

QS12

2. What is your age interval?

QS12=1 ◯ 18~25 years

QS12=2 ◯ 26~30 years

QS12=3 ◯ 31~35 years

QS12=4 ◯ 36~40 years

QS12=5 ◯ 41~45 years

QS12=6 ◯ 46~50 years

QS12=7 ◯ 51~55 years

QS12=8 ◯ 56~60 years

QS12=9 ◯ Above 60 years

QS13

3. What is your highest educational level?

QS13=1 ◯ Pre-primary education or primary school education

QS13=2 ◯ Middle school education

QS13=3 ◯ High school education

QS13=4 ◯ Vocational school education (or other same-level degrees)

QS13=5 ◯ Bachelor's degree (or other same-level degrees)

QS13=6 ◯ Master's degree (or other same-level degrees)

QS13=7 ◯ PhD degree (or other same-level degrees)

QS14

4. What country/region are you currently in? [dropdown]

QS15

5. What is the best description of your occupation or your working area?

QS15=1
Students

QS15=2
Managers

QS15=3
Professionals

QS15=4
Technicians and associate professionals

QS15=5
Clerical support workers

QS15=6
Service and sales workers

QS15=7
Skilled agricultural, forestry and fishery workers

QS15=8
Craft and related trades workers

QS15=9
Plant and machine operators and assemblers

QS15=10
Elementary occupations

QS15=11
Armed forces occupations

QS15=12
QS15_12_other
Other

QS16

6. How much is your annual salary? (Students can skip this question)

QS16=1
Under 10,000 USD per year

QS16=2
10,001-20,000 USD per year

QS16=3
20,001-30,000 USD per year

QS16=4
30,001-40,000 USD per year

QS16=5
40,001-50,000 USD per year

QS16=6
50,001-60,000 USD per year

QS16=7
60,001-70,000 USD per year

QS16=8
Above 70,000 USD per year

QS17

7. How do you rate your willingness and acceptance to get COVID-19 vaccination? (Rate from totally unwilling 0 to totally willing 10, if the vaccines are generally available)

totally unwilling 0	QS17_1=0	QS17_1=1	QS17_1=2	QS17_1=3	QS17_1=4	QS17_1=5	QS17_1=6	QS17_1=7	QS17_1=8	QS17_1=9	QS

QS18

8. How do you rate your willingness and acceptance if your friends, family members or employers recommend that you do so? (Rate from totally unwilling 0 to totally willing 10, if th
generally available)

totally unwilling 0	QS18_1=0	QS18_1=1	QS18_1=2	QS18_1=3	QS18_1=4	QS18_1=5	QS18_1=6	QS18_1=7	QS18_1=8	QS18_1=9	QS

QS19

9. Have you ever been infected with COVID-19?

QS19=1 Yes

QS19=2 No

QS19=3 I don't want to answer.

QS110

10. Have your friends, family members, employers, neighbours, or anybody you know in your community been infected with COVID-19?

QS110=1 Yes

QS110=2 No

QS110=3 I don't want to answer.

QS111

11. What is your marital status?

QS111=1 Single

QS111=2 Married

QS111=3 Divorced

QS111=4 Other

QS111=5 I don't want to answer.

QS112

12. What's your major source of COVID-19 vaccine information?

QS112_1 Healthcare provider

QS112_2 CDC or state or local public health department

QS112_3 News reports (Internet, TV, radio, newspaper)

QS112_4 Social media

QS112_5 Friends and family

QS112_6 Employer

QS112_7 Advertisements from drug companies

QS112_8 QS112_8_other Other

Previous Next

0% 100%

explain

Section 2. Choosing the best answer based on a scenario

Previous Next

0% 100%

CBC_Random1

Scenario#1 Suppose you are going to get vaccinated, and you are facing several choices of action. Please select the answer that you are most satisfied with？

Once you have completed this question, you can still click the back button to return to this page at any time, and you can change your answer at any time.

	Vaccine A	Vaccine B	Neither
Vaccine varieties	Inactivated vaccine	Adenovirus vector vaccines	Neither
Adverse effect	moderate	very mild	
Efficacy	85%	75%	
Time for the vaccine starting working	10 days	5 days	
The duration of vaccine works	10 months	15 months	
The cost of vaccination	$0	$50	
	CBC_Random1<br>Select This	CBC_Random1<br>Select This	CBC_Random1<br>Select This

Previous Next

0% 100%

CBC_Random2

Scenario#2 Suppose you are going to get vaccinated, and you are facing several choices of action. Please select the answer that you are most satisfied with？

Once you have completed this question, you can still click the back button to return to this page at any time, and you can change your answer at any time.

	Vaccine A	Vaccine B	Neither
Vaccine varieties	mRNA vaccine	mRNA vaccine	Neither
Adverse effect	mild	moderate	
Efficacy	65%	55%	
Time for the vaccine starting working	15 days	20 days	
The duration of vaccine works	20 months	5 months	
The cost of vaccination	$150	$0	
	CBC_Random2<br>Select This	CBC_Random2<br>Select This	CBC_Random2<br>Select This

Previous Next

0% 100%

CBC_Random3

Scenario#3 Suppose you are going to get vaccinated, and you are facing several choices of action. Please select the answer that you are most satisfied with?

Once you have completed this question, you can still click the back button to return to this page at any time, and you can change your answer at any time.

	Vaccine A	Vaccine B	Neither
Vaccine varieties	Inactivated vaccine	Adenovirus vector vaccines	Neither
Adverse effect	mild	very mild	
Efficacy	55%	85%	
Time for the vaccine starting working	20 days	10 days	
The duration of vaccine works	15 months	5 months	
The cost of vaccination	$100	$200	
	CBC_Random3 Select This	CBC_Random3 Select This	CBC_Random3 Select This

Previous Next

0% 100%

CBC_Fixed1

Scenario#4 Suppose you are going to get vaccinated, and you are facing several choices of action. Please select the answer that you are most satisfied with？

Once you have completed this question, you can still click the back button to return to this page at any time, and you can change your answer at any time.

	Vaccine A	Vaccine B	Neither
Vaccine varieties	mRNA vaccine	Inactivated vaccine	Neither
Adverse effect	very mild	mild	
Efficacy	65%	85%	
Time for the vaccine starting working	10 days	15 days	
The duration of vaccine works	10 months	20 months	
The cost of vaccination	$100	$50	
	CBC_Fixed1 Select This	CBC_Fixed1 Select This	CBC_Fixed1 Select This

Previous Next

0% 100%

CBC_Random6

Scenario#5 Suppose you are going to get vaccinated, and you are facing several choices of action. Please select the answer that you are most satisfied with?

Once you have completed this question, you can still click the back button to return to this page at any time, and you can change your answer at any time.

	Vaccine A	Vaccine B	Neither
Vaccine varieties	mRNA vaccine	mRNA vaccine	
Adverse effect	mild	very mild	
Efficacy	65%	85%	
Time for the vaccine starting working	5 days	20 days	Neither
The duration of vaccine works	5 months	20 months	
The cost of vaccination	$200	$50	
	CBC_Random6 Select This	CBC_Random6 Select This	CBC_Random6 Select This

Previous Next

0% 100%

CBC_Random4

Scenario#6 Suppose you are going to get vaccinated, and you are facing several choices of action. Please select the answer that you are most satisfied with?

Once you have completed this question, you can still click the back button to return to this page at any time, and you can change your answer at any time.

	Vaccine A	Vaccine B	Neither
Vaccine varieties	Adenovirus vector vaccines	mRNA vaccine	Neither
Adverse effect	mild	moderate	
Efficacy	95%	95%	
Time for the vaccine starting working	5 days	15 days	
The duration of vaccine works	20 months	10 months	
The cost of vaccination	$50	$100	
	CBC_Random4 Select This	CBC_Random4 Select This	CBC_Random4 Select This

Previous Next

0% 100%

CBC_Random5

Scenario#7 Suppose you are going to get vaccinated, and you are facing several choices of action. Please select the answer that you are most satisfied with?

Once you have completed this question, you can still click the back button to return to this page at any time, and you can change your answer at any time.

	Vaccine A	Vaccine B	Neither
Vaccine varieties	Inactivated vaccine	Adenovirus vector vaccines	Neither
Adverse effect	very mild	moderate	
Efficacy	75%	55%	
Time for the vaccine starting working	15 days	10 days	
The duration of vaccine works	15 months	20 months	
The cost of vaccination	$150	$200	
	CBC_Random5 Select This	CBC_Random5 Select This	CBC_Random5 Select This

Previous Next

0% 100%

CBC_Fixed2

Scenario#8 Suppose you are going to get vaccinated, and you are facing several choices of action. Please select the answer that you are most satisfied with?

Once you have completed this question, you can still click the back button to return to this page at any time, and you can change your answer at any time.

	Vaccine A	Vaccine B	Neither
Vaccine varieties	Adenovirus vector vaccines	mRNA vaccine	Neither
Adverse effect	very mild	moderate	
Efficacy	65%	95%	
Time for the vaccine starting working	5 days	15 days	
The duration of vaccine works	10 months	20 months	
The cost of vaccination	$100	$150	
	CBC_Fixed2<br>Select This	CBC_Fixed2<br>Select This	CBC_Fixed2<br>Select This

Previous Next

0% 100%

CBC_Random7

Scenario#9 Suppose you are going to get vaccinated, and you are facing several choices of action. Please select the answer that you are most satisfied with？

Once you have completed this question, you can still click the back button to return to this page at any time, and you can change your answer at any time.

	Vaccine A	Vaccine B	Neither
Vaccine varieties	mRNA vaccine	Inactivated vaccine	Neither
Adverse effect	moderate	mild	
Efficacy	95%	65%	
Time for the vaccine starting working	10 days	20 days	
The duration of vaccine works	15 months	10 months	
The cost of vaccination	$150	$100	
	CBC_Random7 Select This	CBC_Random7 Select This	CBC_Random7 Select This

Previous Next

0% 100%

CBC_Fixed3

Scenario#10 Suppose you are going to get vaccinated, and you are facing several choices of action. Please select the answer that you are most satisfied with？

Once you have completed this question, you can still click the back button to return to this page at any time, and you can change your answer at any time.

	Vaccine A	Vaccine B	Neither
Vaccine varieties	Inactivated vaccine	Adenovirus vector vaccines	Neither
Adverse effect	mild	moderate	
Efficacy	65%	85%	
Time for the vaccine starting working	10 days	15 days	
The duration of vaccine works	10 months	15 months	
The cost of vaccination	$50	$100	
	CBC_Fixed3<br>Select This	CBC_Fixed3<br>Select This	CBC_Fixed3<br>Select This

Previous Next

0% 100%

CBC_Random8

Scenario#11 Suppose you are going to get vaccinated, and you are facing several choices of action. Please select the answer that you are most satisfied with？

Once you have completed this question, you can still click the back button to return to this page at any time, and you can change your answer at any time.

	Vaccine A	Vaccine B	Neither
Vaccine varieties	Adenovirus vector vaccines	Inactivated vaccine	Neither
Adverse effect	mild	very mild	
Efficacy	75%	95%	
Time for the vaccine starting working	15 days	5 days	
The duration of vaccine works	10 months	20 months	
The cost of vaccination	$0	$200	
	CBC_Random8<br>Select This	CBC_Random8<br>Select This	CBC_Random8<br>Select This

Previous Next

0% 100%

Q3

Section 3. Psychological and behavioural part

Q4

This is the last part of our questionnaire. Thank you for your patience and support!

QS31

Past immunization behaviour/ adverse events (Dichotomous; Please answer with yes or no.)

- “I have delayed getting a vaccine shot once (or more than once) for reasons other than illness or allergy."

QS31=1 ◯ Yes

QS31=2 ◯ No

QS32

- “I have once decided not to get a shot of vaccine for reasons other than illness or allergy?”

QS32=1 ◯ Yes

QS32=2 ◯ No

QS33

- “I have events in the past that discouraged me from getting a vaccine(s) for myself or my families?”

QS33=1 ◯ Yes

QS33=2 ◯ No

QS34

- Did you ever experience an AEFI (adverse event following immunization)?

QS34=1 ◯ Yes

QS34=2 ◯ No

QS35

Cues to action (dichotomous, please answer yes/no)

- “I am recommended by a doctor to get COVID-19 vaccination.”

QS35=1 ◯ Yes

QS35=2 ◯ No

QS36

• "I am recommended by the local health board to get COVID-19 vaccination."

QS36=1 Yes

QS36=2 No

QS37

• "I heard that my friends/families are being vaccinated."

QS37=1 Yes

QS37=2 No

Q20

Likert 7-point scales were applied to the following questions. Please rate: totally disagree 1 - totally agree 7

QS38

• "I feel I get enough information about COVID-19 vaccines and their safety."

totally disagree 1	QS38_1=1	QS38_1=2	QS38_1=3	QS38_1=4	QS38_1=5	QS38_1=6	QS38_1=7	totally agree 7

QS39

• "The information I receive about COVID-19 vaccines from the vaccine program is reliable and trustworthy."

totally disagree 1	QS39_1=1	QS39_1=2	QS39_1=3	QS39_1=4	QS39_1=5	QS39_1=6	QS39_1=7	totally agree 7

QS310

• "I think there is a great chance for me to be infected with COVID-19."

totally disagree	QS310_1=1	QS310_1=2	QS310_1=3	QS310_1=4	QS310_1=5	QS310_1=6	QS310_1=7	totally agree 7

QS311

• "I think a COVID-19 infection would be a serious threat to health."

totally disagree 1	QS311_1=1	QS311_1=2	QS311_1=3	QS311_1=4	QS311_1=5	QS311_1=6	QS311_1=7	totally agree 7

Q25

Perceived benefits

QS312

1. Self-protection （please rate: totally disagree 1 - totally agree 7）

• "Getting COVID-19 vaccines is a good way to protect myself from COVID-19."

totally disagree	QS312_1=1	QS312_1=2	QS312_1=3	QS312_1=4	QS312_1=5	QS312_1=6	QS312_1=7	totally agree

1	○	○	○	○	○	○	○	7

QS313

2. Prevent illness in patients (please rate: totally disagree 1 - totally agree 7)

• “Getting COVID-19 vaccines is a good way to prevent COVID-19 infection spread by patients.”

totally disagree 1	QS313_1=1 ○	QS313_1=2 ○	QS313_1=3 ○	QS313_1=4 ○	QS313_1=5 ○	QS313_1=6 ○	QS313_1=7 ○	totally agree 7

QS314

3. Prevent illness in family or friends (please rate: totally disagree 1 - totally agree 7)

• “Getting COVID-19 vaccines is a good way to protect my friends or families from infected by COVID-19."

totally disagree 1	QS314_1=1 ○	QS314_1=2 ○	QS314_1=3 ○	QS314_1=4 ○	QS314_1=5 ○	QS314_1=6 ○	QS314_1=7 ○	totally agree 7

Q29

Perceived risks and barriers

QS315

1. Acquiring the disease (please rate: totally disagree 1 - totally agree 7)

• “I am afraid that COVID-19 vaccine may, in fact, cause me to get infected with COVID-19.”

totally disagree 1	QS315_1=1 ○	QS315_1=2 ○	QS315_1=3 ○	QS315_1=4 ○	QS315_1=5 ○	QS315_1=6 ○	QS315_1=7 ○	totally agree 7

QS316

2. Disease from vaccination (please rate: totally disagree 1 - totally agree 7)

• “I am concerned that I might have a serious side effect from a shot of COVID-19 vaccine.”

tottaly disagree 1	QS316_1=1 ○	QS316_1=2 ○	QS316_1=3 ○	QS316_1=4 ○	QS316_1=5 ○	QS316_1=6 ○	QS316_1=7 ○	totally agree 7

QS317

3. Vaccine unsafe (please rate: totally disagree 1 - totally agree 7)

• “I am concerned that the production, storage, transportation, and unprofessional injection administration may cause the COVID-19 vaccines to be unsafe for taking.”

totally disagree 1	QS317_1=1 ○	QS317_1=2 ○	QS317_1=3 ○	QS317_1=4 ○	QS317_1=5 ○	QS317_1=6 ○	QS317_1=7 ○	totally agree 7

Q33

Perceived safety and efficacy of vaccine

QS318

1. Belief that vaccine is effective (please rate: totally disagree 1 - totally agree 7)

• “I am concerned that taking the vaccines might not prevent COVID-19.”

totally disagree 1	QS318_1=1 ○	QS318_1=2 ○	QS318_1=3 ○	QS318_1=4 ○	QS318_1=5 ○	QS318_1=6 ○	QS318_1=7 ○	totally agree 7

QS319

• "It is better to develop immunity by getting vaccinated than by getting infected with COVID-19."

totally disagree 1	QS319_1=1	QS319_1=2	QS319_1=3	QS319_1=4	QS319_1=5	QS319_1=6	QS319_1=7	totally agree 7
	◯	◯	◯	◯	◯	◯	◯	

QS320

2. Belief that vaccine is safe (please rate: totally disagree 1 - totally agree 7)

• "I believe the COVID-19 vaccine is safe."

totally disagree 1	QS320_1=1	QS320_1=2	QS320_1=3	QS320_1=4	QS320_1=5	QS320_1=6	QS320_1=7	totally agree 7
	◯	◯	◯	◯	◯	◯	◯	

Q37

General attitudes and trust for vaccines (please rate: totally disagree 1 — totally agree 7)

QS321

• "In general, prevention is better than cure."

totally disagree 1	QS321_1=1	QS321_1=2	QS321_1=3	QS321_1=4	QS321_1=5	QS321_1=6	QS321_1=7	totally agree 7
	◯	◯	◯	◯	◯	◯	◯	

QS322

• "In general, vaccination is effective in preventing diseases."

totally disagree 1	QS322_1=1	QS322_1=2	QS322_1=3	QS322_1=4	QS322_1=5	QS322_1=6	QS322_1=7	totally agree 7
	◯	◯	◯	◯	◯	◯	◯	

QS323

• "I trust the information I receive about COVID-19 vaccines."

totally disagree 1	QS323_1=1	QS323_1=2	QS323_1=3	QS323_1=4	QS323_1=5	QS323_1=6	QS323_1=7	totally agree 7
	◯	◯	◯	◯	◯	◯	◯	

QS324

• "I am able to openly discuss my concerns about COVID-19 vaccine shots with my doctor."

totally disagree 1	QS324_1=1	QS324_1=2	QS324_1=3	QS324_1=4	QS324_1=5	QS324_1=6	QS324_1=7	totally agree 7
	◯	◯	◯	◯	◯	◯	◯	

QS325

• "I feel confident that the health centre or doctor's office will have the COVID-19 vaccine when I need to take it."

totally disagree 1	QS325_1=1	QS325_1=2	QS325_1=3	QS325_1=4	QS325_1=5	QS325_1=6	QS325_1=7	totally agree 7
	◯	◯	◯	◯	◯	◯	◯	

QS326

• "I trust that my government is making decisions in my best interest with respect to what COVID-19 vaccines are provided."

totally disagree 1	QS326_1=1	QS326_1=2	QS326_1=3	QS326_1=4	QS326_1=5	QS326_1=6	QS326_1=7	totally agree 7
	◯	◯	◯	◯	◯	◯	◯	

QS327

• "I think COVID-19 vaccines are more important for boys/men" or "I think COVID-19 vaccines are more important for girls/women."

totally disagree 1	QS327_1=1	QS327_1=2	QS327_1=3	QS327_1=4	QS327_1=5	QS327_1=6	QS327_1=7	totally agree 7
	◯	◯	◯	◯	◯	◯	◯	

QS328

• “I decide to take/not to take the COVID-19 vaccine because of religious or cultural reasons.”

totally disagree 1	QS328_1=1	QS328_1=2	QS328_1=3	QS328_1=4	QS328_1=5	QS328_1=6	QS328_1=7	totally agree 7
	◯	◯	◯	◯	◯	◯	◯	

QS329

• “I believe that people are risking their health or the health of the society if they do not take a COVID-19 vaccine."

totally disagree 1	QS329_1=1	QS329_1=2	QS329_1=3	QS329_1=4	QS329_1=5	QS329_1=6	QS329_1=7	totally agree 7
	◯	◯	◯	◯	◯	◯	◯	

QS330

• “I think it is important for everyone to get the recommended COVID-19 vaccines for themselves.”

totally disagree 1	QS330_1=1	QS330_1=2	QS330_1=3	QS330_1=4	QS330_1=5	QS330_1=6	QS330_1=7	totally agree 7
	◯	◯	◯	◯	◯	◯	◯	

QS331

• “It’s important for me to spend more than one hour in travel time to get a COVID-19 vaccine.”

totally disagree 1	QS331_1=1	QS331_1=2	QS331_1=3	QS331_1=4	QS331_1=5	QS331_1=6	QS331_1=7	totally agree 7
	◯	◯	◯	◯	◯	◯	◯	

QS332

• “I believe that COVID-19 vaccine producers care about my health more than their profit.”

totally disagree 1	QS332_1=1	QS332_1=2	QS332_1=3	QS332_1=4	QS332_1=5	QS332_1=6	QS332_1=7	totally agree 7
	◯	◯	◯	◯	◯	◯	◯	

Previous Next

0% 100%

QSP

Thanks again for your support! But we have seven more scenarios that also need your help! Thanks!

Previous Next

0% 100%

CBC2_Random1

Scenario#1 When you get a fever, and you don't know whether you have been infected with COVID-19， please select the one medical diagnosis that you feel the most satisfied with？

If you have answered this question, you can still click the back button to return to this page at any time, and you can change your answer at any time.

	Service 1	Service 2	Neither
Clinics	Network consultation	Outpatient fever clinic	Neither.
Staff	Nurse	Paramedic	
Waiting time	75 min	15 min	
Immediate diagnostic COVID-19 tests	Yes	No	
Diagnosis/consultation expenses	0 USD	75 USD	
Reimbursement rate/Claims	20%	80%	
	CBC2_Random1 Select This	CBC2_Random1 Select This	CBC2_Random1 Select This

Previous Next

0% 100%

CBC2_Fixed1

Scenario#2 When you get a fever, and you don't know whether you have been infected with COVID-19， please select the one medical diagnosis that you feel the most satisfied with？

If you have answered this question, you can still click the back button to return to this page at any time, and you can change your answer at any time.

	Service 1	Service 2	Neither
Clinics	Telephone consultation	Private patient clinic	Neither.
Staff	Nurse	Doctor	
Waiting time	0 min	30 min	
Immediate diagnostic COVID-19 tests	No	Yes	
Diagnosis/consultation expenses	25 USD	75 USD	
Reimbursement rate/Claims	20%	60%	
	CBC2_Fixed1	CBC2_Fixed1	CBC2_Fixed1
	Select This	Select This	Select This

Previous Next

0% 100%

CBC2_Random2

Scenario#3 When you get a fever, and you don't know whether you have been infected with COVID-19， please select the one medical diagnosis that you feel the most satisfied with？

If you have answered this question, you can still click the back button to return to this page at any time, and you can change your answer at any time.

	Service 1	Service 2	Neither
Clinics	Telephone consultation	Emergency department	Neither.
Staff	Paramedic	Doctor	
Waiting time	60 min	45 min	
Immediate diagnostic COVID-19 tests	Yes	No	
Diagnosis/consultation expenses	0 USD	50 USD	
Reimbursement rate/Claims	0%	100%	
	CBC2_Random2 Select This	CBC2_Random2 Select This	CBC2_Random2 Select This

Previous Next

0% 100%

CBC2_Random3

Scenario#4 When you get a fever, and you don't know whether you have been infected with COVID-19， please select the one medical diagnosis that you feel the most satisfied with？

If you have answered this question, you can still click the back button to return to this page at any time, and you can change your answer at any time.

	Service 1	Service 2	Neither
Clinics	Private patient clinic	Telephone consultation	Neither.
Staff	Paramedic	Doctor	
Waiting time	0 min	30 min	
Immediate diagnostic COVID-19 tests	Yes	No	
Diagnosis/consultation expenses	25 USD	25 USD	
Reimbursement rate/Claims	60%	40%	
	CBC2_Random3 Select This	CBC2_Random3 Select This	CBC2_Random3 Select This

Previous Next

0% 100%

CBC2_Random4

Scenario#5 When you get a fever, and you don't know whether you have been infected with COVID-19， please select the one medical diagnosis that you feel the most satisfied with？

If you have answered this question, you can still click the back button to return to this page at any time, and you can change your answer at any time.

	Service 1	Service 2	Neither
Clinics	Private patient clinic	Network consultation	Neither.
Staff	Nurse	Doctor	
Waiting time	60 min	30 min	
Immediate diagnostic COVID-19 tests	No	Yes	
Diagnosis/consultation expenses	100 USD	75 USD	
Reimbursement rate/Claims	80%	60%	
	CBC2_Random4	CBC2_Random4	CBC2_Random4
	Select This	Select This	Select This

Previous Next

0% 100%

CBC2_Random5

Scenario#6 When you get a fever, and you don't know whether you have been infected with COVID-19， please select the one medical diagnosis that you feel the most satisfied with？

If you have answered this question, you can still click the back button to return to this page at any time, and you can change your answer at any time.

	Service 1	Service 2	Neither
Clinics	Emergency department	Outpatient fever clinic	Neither.
Staff	Nurse	Nurse	
Waiting time	0 min	75 min	
Immediate diagnostic COVID-19 tests	Yes	Yes	
Diagnosis/consultation expenses	100 USD	50 USD	
Reimbursement rate/Claims	20%	0%	
	CBC2_Random5	CBC2_Random5	CBC2_Random5
	Select This	Select This	Select This

Previous Next

0% 100%

CBC2_Random6

Scenario#7 When you get a fever, and you don't know whether you have been infected with COVID-19， please select the one medical diagnosis that you feel the most satisfied with？

If you have answered this question, you can still click the back button to return to this page at any time, and you can change your answer at any time.

	Service 1	Service 2	Neither
Clinics	Outpatient fever clinic	Private patient clinic	Neither.
Staff	Doctor	Paramedic	
Waiting time	15 min	45 min	
Immediate diagnostic COVID-19 tests	Yes	No	
Diagnosis/consultation expenses	25 USD	100 USD	
Reimbursement rate/Claims	100%	40%	
	CBC2_Random6 Select This	CBC2_Random6 Select This	CBC2_Random6 Select This

Previous Next

0% 100%

Q50

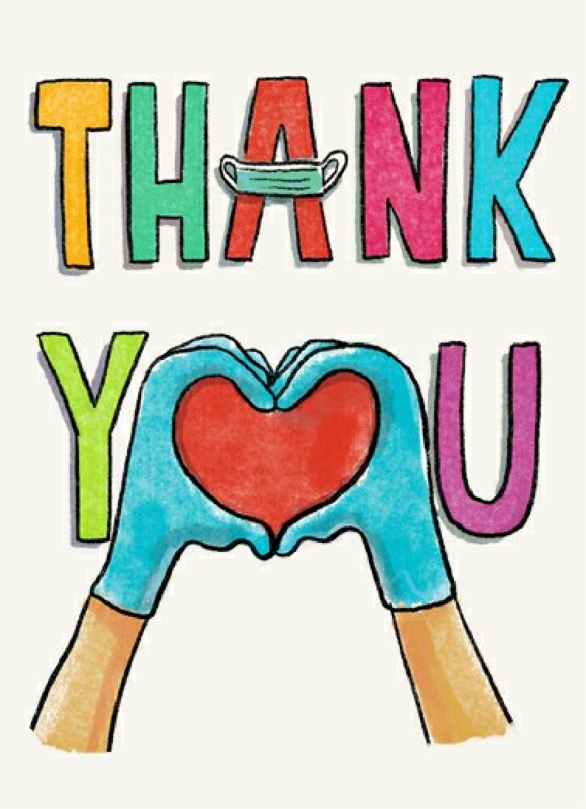

Thank you for your supporting! If you feel interested in our research and want to deeply join in our research, you can send email to t.liu.10@student.rug.nl, we will be looking forward to your participation! And we will randomly send out secrete rewards!

COVID-19 Vaccinaton Willingness Research Team

[Script]

Previous Next

0% 100%

end1

Thanks !

[Script]

0% 100%
