# Supplementary material for: Preferences for Attributes of Initial COVID-19 Diagnosis in the United States and China During the Pandemic: Discrete Choice Experiment With Propensity Score Matching
Source: JMIR Public Health Surveill. 2022 Aug 16;8(8):e37422. doi: 10.2196/37422 (PMC9384860; doi:10.2196/37422)
Supplement: Multimedia Appendix 2 [file publichealth_v8i8e37422_app2.pdf]

Start

Title

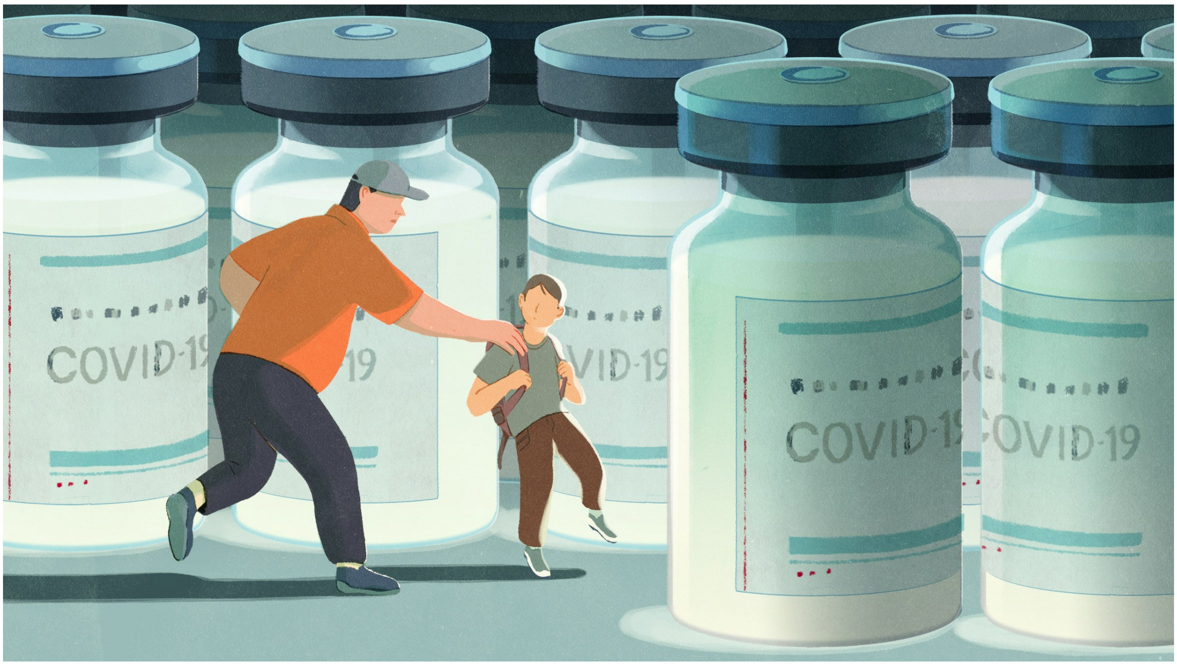

新冠肺炎疫苗的接种意愿和接受度调查问卷

Intro

背景介绍

新冠肺炎疫情对全球社会和经济产生了深远影响。 科学家和制药公司一直在研究新冠肺炎的疫苗。 有关几种疫苗的报告已经发布，一些国家已经启动了疫苗接种计划。

这项调查旨在调查公众对新冠肺炎疫苗接种的意愿和接受程度。

下一页

Preface

前言

感谢您的参与！ 该问卷包括3部分问题，可以在大约10分钟内完成。 您可以随时退出此调查表。 我们将对您的信息保密。

后退 下一页

0% 100%

Consent

您好,

您即将参加我们的问卷调查。 该调查问卷将包括几个部分，只需不超过10分钟即可完成。 您同意接受问卷调查吗？

Consent=1 ◯ 同意

Consent=2 ◯ 不同意

AgeConsent

您是否大于18岁?

AgeConsent=1 ◯ 是

AgeConsent=2 ◯ 否

该研究不包括生物医学公司的任何资金。 如有任何疑问，请随时发送电子邮件至：t.liu.10@student.rug.nl 或 mingwj@jnu.edu.cn

暨南大学基础医学与公共卫生学院 新冠病毒疫苗接种意愿研究组

陕西（高校）哲学社会科学重点研究基地-健康文化研究中心

后退 下一页

0% 100%

Q1

第一节. 基本信息

QS11

1. 您的性别是?

QS11=1 ◯ 男性

QS11=2 ◯ 女性

QS11=3 ◯ 其他

QS12

2. 您的年龄区间是?

QS12=1 ◯ 18~25岁

QS12=2 ◯ 26~30岁

QS12=3 ◯ 31~35岁

QS12=4 ◯ 36~40岁

QS12=5 ◯ 41~45岁

QS12=6 ◯ 46~50岁

QS12=7 ◯ 51~55岁

QS12=8 ◯ 56~60岁

QS12=9 ◯ 大于60岁

QS13

3. 您的最高学历是?

QS13=1 ◯ 学前教育或小学教育

QS13=2 ◯ 初级中学教育

QS13=3 ◯ 高级中学教育

QS13=4 ◯ 职业学校教育（或其他同等学位）

QS13=5 ◯ 学士学位（或其他同等学位）

QS13=6 ◯ 硕士学位（或其他同等学位）

QS13=7 ◯ 博士学位（或其他同等学位）

QS15

4. 对您的职业或工作领域的最佳描述是什么？

QS15=1
◯ 学生

QS15=2
◯ 管理人员

QS15=3
◯ 专业人士

QS15=4
◯ 技术人员和助理专业人员

QS15=5
◯ 文书支持人员

QS15=6
◯ 服务和销售人员

QS15=7
◯ 农业，林业和渔业技术工人

QS15=8
◯ 工艺及相关行业工人

QS15=9
◯ 工厂和机器操作员及装配工

QS15=10
◯ 初级岗位（如清洁，擦洗等）

QS15=11
◯ 武装部队职业

QS15=12
◯ QS15_12_other
其他 ______

QS16

5. 你的年薪是？（学生可以跳过此问题）

QS16=1
◯ 低于64267元/年

QS16=2
◯ 64273-128534元/年

QS16=3
◯ 128541-192802元/年

QS16=4
◯ 192808-257069元/年

QS16=5
◯ 257075-321336元/年

QS16=6
◯ 321343-385604年/年

QS16=7
◯ 385610-449871元/年

QS16=8
◯ 大于449871元/年

QS17

6. 您如何评价自己接受新冠病毒疫苗疫苗的意愿和接受程度？（假设通常可以接种疫苗的情况下，从完全不愿意0分到完全愿意10分）

完全不愿意0	QS17_1=0	QS17_1=1	QS17_1=2	QS17_1=3	QS17_1=4	QS17_1=5	QS17_1=6	QS17_1=7	QS17_1=8	QS17_1=9	QS1
	◯	◯	◯	◯	◯	◯	◯	◯	◯	◯	

QS18

7. 如果您的朋友，家人或雇主建议您接种疫苗，您如何评价自己对于接种新冠病毒疫苗的意愿和接受程度？（假设通常可以接种疫苗的情况下，从完全
完全愿意10分）

完全

不愿意 0	QS18_1=0	QS18_1=1	QS18_1=2	QS18_1=3	QS18_1=4	QS18_1=5	QS18_1=6	QS18_1=7	QS18_1=8	QS18_1=9	QS1
	◯	◯	◯	◯	◯	◯	◯	◯	◯	◯	

QS19

8. 您是否曾经感染过新冠病毒?

QS19=1 ◯ 是

QS19=2 ◯ 否

QS19=3 ◯ 我不想回答

QS110

9. 您的朋友，家人，雇主，邻居或某个您在社区中认识的人是否曾经感染过新冠病毒?

QS110=1 ◯ 是

QS110=2 ◯ 否

QS110=3 ◯ 我不想回答

QS111

10. 您的婚姻状况是?

QS111=1 ◯ 未婚

QS111=2 ◯ 已婚

QS111=3 ◯ 离异

QS111=4 ◯ 其他

QS111=5 ◯ 我不想回答

QS112

11. 您通过何种渠道获取新冠病毒疫苗的相关信息?（可多选）

QS112_1 ☐ 医疗保健行业

QS112_2 ☐ 疾病预防控制中心或地方公共卫生部门

QS112_3 ☐ 新闻报道（互联网，电视，广播，报纸）

QS112_4 ☐ 社交媒体

QS112_5 ☐ 朋友或家人

QS112_6 ☐ 雇主（上司）

QS112_7 ☐ 医药公司的广告

QS112_8 ☐ QS112_8_other 其他 ____

后退 下一页

0% 100%

explain

第2节. 根据场景选择最佳答案

您将会被问到11个类似的情景题，请细心阅读并选择您更倾向的情景。

后退 下一页

0% 100%

CBC_Random1

场景#1/11个场景 当您准备接种新冠疫苗时，您可能会面临以下几种关于疫苗的情景，某些情景可能并不符合现实情况，但也请您根据**假设**情况以及现有信息，从以下三个选项中选择您最满意的一种情景。

完成此问题后，您仍然可以随时单击“后退”按钮以随时返回此页面，并且可以随时更改答案。将鼠标移到疫苗属性的上方可以查看相关提示。

	疫苗 A	疫苗 B	都不选
疫苗种类	灭活疫苗	腺病毒载体疫苗	都不选
副作用	中度症状	轻微症	
有效性	85%	75%	
疫苗起作用时间	10 天	5 天	
疫苗有效时间	10个月	15个月	
疫苗接种费用	0元	320元	
	CBC_Random1	CBC_Random1	CBC_Random1
	选择此项	选择此项	选择此项

后退 下一页

0% 100%

CBC_Random2

场景#2/11个场景 当您准备接种新冠疫苗时，您可能会面临以下几种关于疫苗的情景，某些情景可能并不符合现实情况，但也请您根据**假设**情况以及现有信息，从以下三个选项中选择您最满意的一种情景。

完成此问题后，您仍然可以随时单击“后退”按钮以随时返回此页面，并且可以随时更改答案。将鼠标移到疫苗属性的上方可以查看相关提示。

	疫苗 A	疫苗 B	都不选
疫苗种类	mRNA疫苗	mRNA疫苗	都不选
副作用	轻症	中度症状	
有效性	65%	55%	
疫苗起作用时间	15 天	20 天	
疫苗有效时间	20个月	5个月	
疫苗接种费用	960元	0元	
	CBC_Random2 选择此项	CBC_Random2 选择此项	CBC_Random2 选择此项

后退 下一页

0% 100%

CBC_Random3

场景#3/11个场景 当您准备接种新冠疫苗时，您可能会面临以下几种关于疫苗的情景，某些情景可能并不符合合现实情况，但也请您根据**假设**情况以及现有信息，从以下三个选项中选择您最满意的一种情景。

完成此问题后，您仍然可以随时单击“后退”按钮以随时返回此页面，并且可以随时更改答案。将鼠标移到疫苗属性的上方可以查看相关提示。

	疫苗 A	疫苗 B	都不选
疫苗种类	灭活疫苗	腺病毒载体疫苗	都不选
副作用	轻症	轻微症	
有效性	55%	85%	
疫苗起作用时间	20 天	10 天	
疫苗有效时间	15个月	5个月	
疫苗接种费用	640元	1280元	
	CBC_Random3 选择此项	CBC_Random3 选择此项	CBC_Random3 选择此项

后退 下一页

0% 100%

CBC_Fixed1

场景#4/11个场景 当您准备接种新冠疫苗时，您可能会面临以下几种关于疫苗的情景，某些情景可能并不符合现实情况，但也请您根据**假设**情况以及现有信息，从以下三个选项中选择您最满意的一种情景。

完成此问题后，您仍然可以随时单击“后退”按钮以随时返回此页面，并且可以随时更改答案。将鼠标移到疫苗属性的上方可以查看相关提示。

	疫苗 A	疫苗 B	都不选
疫苗种类	mRNA疫苗	灭活疫苗	都不选
副作用	轻微症	轻症	
有效性	65%	85%	
疫苗起作用时间	10 天	15 天	
疫苗有效时间	10个月	20个月	
疫苗接种费用	640元	320元	
	CBC_Fixed1	CBC_Fixed1	CBC_Fixed1
	选择此项	选择此项	选择此项

后退 下一页

0% 100%

CBC_Random6

场景#5/11个场景 当您准备接种新冠疫苗时，您可能会面临以下几种关于疫苗的情景，某些情景可能并不符合现实情况，但也请您根据**假设**情况以及现有信息，从以下三个选项中选择您最满意的一种情景。

完成此问题后，您仍然可以随时单击“后退”按钮以随时返回此页面，并且可以随时更改答案。将鼠标移到疫苗属性的上方可以查看相关提示。

	疫苗 A	疫苗 B	都不选
疫苗种类	mRNA疫苗	mRNA疫苗	都不选
副作用	轻症	轻微症	
有效性	65%	85%	
疫苗起作用时间	5 天	20 天	
疫苗有效时间	5个月	20个月	
疫苗接种费用	1280元	320元	
	CBC_Random6<br>选择此项	CBC_Random6<br>选择此项	CBC_Random6<br>选择此项

后退 下一页

0% 100%

CBC_Random4

场景#6/11个场景 当您准备接种新冠疫苗时，您可能会面临以下几种关于疫苗的情景，某些情景可能并不符合现实情况，但也请您根据**假设**情况以及现有信息，从以下三个选项中选择您最满意的一种情景。

完成此问题后，您仍然可以随时单击“后退”按钮以随时返回此页面，并且可以随时更改答案。将鼠标移到疫苗属性的上方可以查看相关提示。

	疫苗 A	疫苗 B	都不选
疫苗种类	腺病毒载体疫苗	mRNA疫苗	都不选
副作用	轻症	中度症状	
有效性	95%	95%	
疫苗起作用时间	5 天	15 天	
疫苗有效时间	20个月	10个月	
疫苗接种费用	320元	640元	
	CBC_Random4 选择此项	CBC_Random4 选择此项	CBC_Random4 选择此项

后退 下一页

0% 100%

CBC_Random5

场景#7/11个场景 当您准备接种新冠疫苗时，您可能会面临以下几种关于疫苗的情景，某些情景可能并不符合现实情况，但也请您根据**假设**情况以及现有信息，从以下三个选项中选择您最满意的一种情景。

完成此问题后，您仍然可以随时单击“后退”按钮以随时返回此页面，并且可以随时更改答案。将鼠标移到疫苗属性的上方可以查看相关提示。

	疫苗 A	疫苗 B	都不选
疫苗种类	灭活疫苗	腺病毒载体疫苗	都不选
副作用	轻微症	中度症状	
有效性	75%	55%	
疫苗起作用时间	15 天	10 天	
疫苗有效时间	15个月	20个月	
疫苗接种费用	960元	1280元	
	CBC_Random5	CBC_Random5	CBC_Random5
	选择此项	选择此项	选择此项

后退 下一页

0% 100%

CBC_Fixed2

场景#8/11个场景 当您准备接种新冠疫苗时，您可能会面临以下几种关于疫苗的情景，某些情景可能并不符合合现实情况，但也请您根据**假设**情况以及现有信息，从以下三个选项中选择您最满意的一种情景。

完成此问题后，您仍然可以随时单击“后退”按钮以随时返回此页面，并且可以随时更改答案。将鼠标移到疫苗属性的上方可以查看相关提示。

	疫苗 A	疫苗 B	都不选
疫苗种类	腺病毒载体疫苗	mRNA疫苗	都不选
副作用	轻微症	中度症状	
有效性	65%	95%	
疫苗起作用时间	5 天	15 天	
疫苗有效时间	10个月	20个月	
疫苗接种费用	640元	960元	
	CBC_Fixed2	CBC_Fixed2	CBC_Fixed2
	选择此项	选择此项	选择此项

后退 下一页

0% 100%

CBC_Random7

场景#9/11个场景 当您准备接种新冠疫苗时，您可能会面临以下几种关于疫苗的情景，某些情景可能并不符合现实情况，但也请您根据**假设**情况以及现有信息，从以下三个选项中选择您最满意的一种情景。

完成此问题后，您仍然可以随时单击“后退”按钮以随时返回此页面，并且可以随时更改答案。将鼠标移到疫苗属性的上方可以查看相关提示。

	疫苗 A	疫苗 B	都不选
疫苗种类	mRNA疫苗	灭活疫苗	都不选
副作用	中度症状	轻症	
有效性	95%	65%	
疫苗起作用时间	10 天	20 天	
疫苗有效时间	15个月	10个月	
疫苗接种费用	960元	640元	
	CBC_Random7 选择此项	CBC_Random7 选择此项	CBC_Random7 选择此项

后退 下一页

0% 100%

CBC_Fixed3

场景#10/11个场景 当您准备接种新冠疫苗时，您可能会面临以下几种关于疫苗的情景，某些情景可能并不符合现实情况，但也请您根据**假设**情况以及现有信息，从以下三个选项中选择您最满意的一种情景。

完成此问题后，您仍然可以随时单击“后退”按钮以随时返回此页面，并且可以随时更改答案。将鼠标移到疫苗属性的上方可以查看相关提示。

	疫苗 A	疫苗 B	都不选
疫苗种类	灭活疫苗	腺病毒载体疫苗	都不选
副作用	轻症	中度症状	
有效性	65%	85%	
疫苗起作用时间	10 天	15 天	
疫苗有效时间	10个月	15个月	
疫苗接种费用	320元	640元	
	CBC_Fixed3 选择此项	CBC_Fixed3 选择此项	CBC_Fixed3 选择此项

后退 下一页

0% 100%

CBC_Random8

场景#11/11个场景 当您准备接种新冠疫苗时，您可能会面临以下几种关于疫苗的情景，某些情景可能并不符合现实情况，但也请您根据**假设**情况以及现有信息，从以下三个选项中选择您最满意的一种情景。

完成此问题后，您仍然可以随时单击“后退”按钮以随时返回此页面，并且可以随时更改答案。将鼠标移到疫苗属性的上方可以查看相关提示。

	疫苗 A	疫苗 B	都不选
疫苗种类	**腺病毒载体疫苗**	**灭活疫苗**	**都不选**
副作用	**轻症**	**轻微症**	
有效性	**75%**	**95%**	
疫苗起作用时间	**15 天**	**5 天**	
疫苗有效时间	**10个月**	**20个月**	
疫苗接种费用	**0元**	**1280元**	
	CBC_Random8	CBC_Random8	CBC_Random8
	选择此项	选择此项	选择此项

后退 下一页

0% 100%

Q3

第三节. 心理和行为部分

Q4

这是我们问卷的最后一部分。 感谢您的耐心配合和支持!

QS31

过去的免疫行为/不良事件（请回答是或否。）

• “我曾经有过一次（或不止一次）因为与生病或过敏无关的其它原因而推迟注射疫苗的经历。”

QS31=1 ◯ 是

QS31=2 ◯ 否

QS32

• “我曾经有过一次（或不止一次）因为与生病或过敏无关的其它原因而决定不注射疫苗的经历。”

QS32=1 ◯ 是

QS32=2 ◯ 否

QS33

• “曾经发生过让我决定不愿再为自己或家人接种疫苗的事件。”

QS33=1 ◯ 是

QS33=2 ◯ 否

QS34

• 您是否曾经经历过预防接种副反应 （即免疫接种后发生的异常反应）?

QS34=1 ◯ 是

QS34=2 ◯ 否

QS35

行动指示（请回答是/否）

• “医生建议我接种新冠病毒疫苗。”

QS35=1 ◯ 是

QS35=2 ◯ 否

QS36

• “当地卫生机构建议我接种新冠病毒的疫苗。”

QS36=1 是

QS36=2 否

QS37

• “我听说我的朋友/家人正在接种新冠病毒的疫苗。”

QS37=1 是

QS37=2 否

Q20

利克特7点量表适用于以下问题。 请评分：从完全不同意 1分-完全同意 7分

QS38

• “我觉得我已获得有关新冠疫苗及其安全性的足够的信息。”

完全不同意 1	QS38_1=1	QS38_1=2	QS38_1=3	QS38_1=4	QS38_1=5	QS38_1=6	QS38_1=7	完全同意 7

QS39

• “我获取到的有关新冠病毒疫苗的信息是可靠且值得信赖的。”

完全不同意 1	QS39_1=1	QS39_1=2	QS39_1=3	QS39_1=4	QS39_1=5	QS39_1=6	QS39_1=7	完全同意 7

QS310

• “我认为我很有可能感染新冠病毒。”

完全不同意 1	QS310_1=1	QS310_1=2	QS310_1=3	QS310_1=4	QS310_1=5	QS310_1=6	QS310_1=7	完全同意 7

QS311

• “我认为感染新冠病毒将严重威胁人体健康。”

完全不同意 1	QS311_1=1	QS311_1=2	QS311_1=3	QS311_1=4	QS311_1=5	QS311_1=6	QS311_1=7	完全同意 7

Q25

感知利益

QS312

1.自我保护（请回答：完全不同意 1分-完全同意 7分）

• “接种新冠病毒疫苗是保护自己免受新冠病毒侵害的好方法。”

完全不同	QS312_1=1	QS312_1=2	QS312_1=3	QS312_1=4	QS312_1=5	QS312_1=6	QS312_1=7	完全同意

意 1	○	○	○	○	○	○	○	7

QS313

2.预防患者传播病毒（请评分：完全不同意 1分-完全同意 7分）

•“接种新冠病毒疫苗是防止新冠肺炎患者传播病毒的好方法。”

完全不同意 1	QS313_1=1 ○	QS313_1=2 ○	QS313_1=3 ○	QS313_1=4 ○	QS313_1=5 ○	QS313_1=6 ○	QS313_1=7 ○	完全同意 7

QS314

3.预防家人或朋友患病（请评分：完全不同意 1分-完全同意 7分）

•“接种新冠病毒疫苗是保护我的朋友或家人免受新冠病毒感染的好方法。”

完全不同意 1	QS314_1=1 ○	QS314_1=2 ○	QS314_1=3 ○	QS314_1=4 ○	QS314_1=5 ○	QS314_1=6 ○	QS314_1=7 ○	完全同意 7

Q29

感知风险和障碍

QS315

1. 疫苗安全性.（请评分：完全不同意 1分-完全同意 7分）

•“我担心接种新冠病毒的疫苗反而会导致我感染新冠病毒。”

完全不同意 1	QS315_1=1 ○	QS315_1=2 ○	QS315_1=3 ○	QS315_1=4 ○	QS315_1=5 ○	QS315_1=6 ○	QS315_1=7 ○	完全同意 7

QS316

2. 接种疫苗引起的副作用.（请评分：完全不同意 1分-完全同意 7分）

•“我担心注射新冠病毒疫苗会对我产生严重的副作用。”

完全不同意 1	QS316_1=1 ○	QS316_1=2 ○	QS316_1=3 ○	QS316_1=4 ○	QS316_1=5 ○	QS316_1=6 ○	QS316_1=7 ○	完全同意 7

QS317

3. 疫苗运输及储存安全性. （请评分：完全不同意 1分-完全同意 7分）

•“我担心疫苗的生产，储存，运输和非专业注射管理等环节可能会导致新冠病毒疫苗的不安全性。”

完全不同意 1	QS317_1=1 ○	QS317_1=2 ○	QS317_1=3 ○	QS317_1=4 ○	QS317_1=5 ○	QS317_1=6 ○	QS317_1=7 ○	完全同意 7

Q33

疫苗的安全性和有效性

QS318

1.疫苗有效信任度（请回答：完全不同意 1分-完全同意 7分）

•“我担心接种新冠病毒疫苗可能无法有效预防新冠病毒。”

完全不同意 1	QS318_1=1 ○	QS318_1=2 ○	QS318_1=3 ○	QS318_1=4 ○	QS318_1=5 ○	QS318_1=6 ○	QS318_1=7 ○	完全同意 7

QS319

•“接种新冠疫苗是比感染新冠病毒更好的获得免疫力的方法。”

完全不同意 1	QS319_1=1 ○	QS319_1=2 ○	QS319_1=3 ○	QS319_1=4 ○	QS319_1=5 ○	QS319_1=6 ○	QS319_1=7 ○	完全同意 7

QS320

2.疫苗是安全的信任度（请评分：完全不同意 1分-完全同意 7分）

• "我相信新冠病毒的疫苗是安全的。"

完全不同意 1	QS320_1=1 ○	QS320_1=2 ○	QS320_1=3 ○	QS320_1=4 ○	QS320_1=5 ○	QS320_1=6 ○	QS320_1=7 ○	完全同意 7

Q37

对疫苗的一般态度和信任度（请回答：完全不同意 1分-完全同意 7分）

QS321

• "总的来说，预防胜于治疗。"

完全不同意 1	QS321_1=1 ○	QS321_1=2 ○	QS321_1=3 ○	QS321_1=4 ○	QS321_1=5 ○	QS321_1=6 ○	QS321_1=7 ○	完全同意 7

QS322

• "总的来说，接种疫苗可以有效预防疾病。"

完全不同意 1	QS322_1=1 ○	QS322_1=2 ○	QS322_1=3 ○	QS322_1=4 ○	QS322_1=5 ○	QS322_1=6 ○	QS322_1=7 ○	完全同意 7

QS323

• "我相信我所获取到的有关新冠病毒疫苗的信息。"

完全不同意 1	QS323_1=1 ○	QS323_1=2 ○	QS323_1=3 ○	QS323_1=4 ○	QS323_1=5 ○	QS323_1=6 ○	QS323_1=7 ○	完全同意 7

QS324

• "我能够与医生公开讨论我对接种新冠病毒疫苗的担忧。"

完全不同意 1	QS324_1=1 ○	QS324_1=2 ○	QS324_1=3 ○	QS324_1=4 ○	QS324_1=5 ○	QS324_1=6 ○	QS324_1=7 ○	完全同意 7

QS325

• "我有信心在我需要接种新冠病毒疫苗时，当地卫生疾控中心或医院有足够的疫苗存量。"

完全不同意 1	QS325_1=1 ○	QS325_1=2 ○	QS325_1=3 ○	QS325_1=4 ○	QS325_1=5 ○	QS325_1=6 ○	QS325_1=7 ○	完全同意 7

QS326

• "我相信我的政府，正在就提供哪种新冠病毒疫苗的问题，而做出符合我最大利益的决定。"

完全不同意 1	QS326_1=1 ○	QS326_1=2 ○	QS326_1=3 ○	QS326_1=4 ○	QS326_1=5 ○	QS326_1=6 ○	QS326_1=7 ○	完全同意 7

QS327

• "我认为新冠病毒疫苗对男孩/男人更重要"或"我认为新冠病毒疫苗对女孩/妇女更重要。"

完全不同意 1	QS327_1=1	QS327_1=2	QS327_1=3	QS327_1=4	QS327_1=5	QS327_1=6	QS327_1=7	完全同意 7

QS328

• “由于宗教或文化原因，我决定接种/不接种新冠病毒疫苗。”

完全不同意 1	QS328_1=1	QS328_1=2	QS328_1=3	QS328_1=4	QS328_1=5	QS328_1=6	QS328_1=7	完全同意 7

QS329

• “我相信，如果不接种新冠病毒疫苗，人们将冒着自己的健康或社会健康的风险。”

完全不同意 1	QS329_1=1	QS329_1=2	QS329_1=3	QS329_1=4	QS329_1=5	QS329_1=6	QS329_1=7	完全同意 7

QS330

• “我认为每个人都能获得官方建议和推荐接种的新冠疫苗很重要。”

完全不同意 1	QS330_1=1	QS330_1=2	QS330_1=3	QS330_1=4	QS330_1=5	QS330_1=6	QS330_1=7	完全同意 7

QS331

• “对于我来说，花一小时以上的时间来获取新冠病毒疫苗对我来说是值得的。”

完全不同意 1	QS331_1=1	QS331_1=2	QS331_1=3	QS331_1=4	QS331_1=5	QS331_1=6	QS331_1=7	完全同意 7

QS332

• “我相信新冠病毒疫苗生产商关心我的健康胜于关心他们的利益。”

完全不同意 1	QS332_1=1	QS332_1=2	QS332_1=3	QS332_1=4	QS332_1=5	QS332_1=6	QS332_1=7	完全同意 7

后退 下一页

0% 100%

QSP

再次感谢您的支持！ 但是我们还有七个问题，也需要您的帮助！ 谢谢！

后退 下一页

0% 100%

CBC2_Random1

情景#1/7个情景 **假设**当您正在经历一次发烧，但是您不确定您是否感染了新冠病毒。您决定寻求诊疗机构的帮助。您可能会面临以下几种关于诊疗服务的情景，某些情景可能并不符合现实情况，但也请您根据**假设**情况以及现有信息，从以下三个选项中选择您最满意的一种情景。

完成此问题后，您仍然可以随时单击“后退”按钮以随时返回此页面，并且可以随时更改答案。将鼠标移到疫苗属性的上方可以查看相关提示。

	诊疗服务 1	诊疗服务 2	都不选
医疗机构	网络咨询	医院发热门诊	都不选.
接诊人员	护士	辅助护理人员	
等待时间	75分钟	15分钟	
立即核酸检测	是	否	
诊断费用	0元	450元	
报销比率	20%	80%	
	CBC2_Random1	CBC2_Random1	CBC2_Random1
	选择此项	选择此项	选择此项

后退 下一页

0% 100%

CBC2_Fixed1

情景#2/7个情景 **假设**当您正在经历一次发烧，但是您不确定您是否感染了新冠病毒。您决定寻求诊疗机构的帮助。您可能会面临以下几种关于诊疗服务的情景，某些情景可能并不符合现实情况，但也请您根据**假设**情况以及现有信息，从以下三个选项中选择您最满意的一种情景。

完成此问题后，您仍然可以随时单击“后退”按钮以随时返回此页面，并且可以随时更改答案。将鼠标移到疫苗属性的上方可以查看相关提示。

	诊疗服务 1	诊疗服务 2	都不选
医疗机构	电话咨询	私人医疗机构	都不选.
接诊人员	护士	医生	
等待时间	0分钟	30分钟	
立即核酸检测	否	是	
诊断费用	150元	450元	
报销比率	20%	60%	
	CBC2_Fixed1 选择此项	CBC2_Fixed1 选择此项	CBC2_Fixed1 选择此项

后退 下一页

0% 100%

CBC2_Random2

情景#3/7个情景 **假设**当您正在经历一次发烧，但是您不确定您是否感染了新冠病毒。您决定寻求诊疗机构的帮助。您可能会面临以下几种关于诊疗服务的情景，某些情景可能并不符合现实情况，但也请您根据**假设**情况以及现有信息，从以下三个选项中选择您最满意的一种情景。

完成此问题后，您仍然可以随时单击“后退”按钮以随时返回此页面，并且可以随时更改答案。将鼠标移到疫苗属性的上方可以查看相关提示。

	诊疗服务 1	诊疗服务 2	都不选
医疗机构	电话咨询	医院急诊部门	都不选.
接诊人员	辅助护理人员	医生	
等待时间	60分钟	45分钟	
立即核酸检测	是	否	
诊断费用	0元	300元	
报销比率	0%	100%	
	CBC2_Random2	CBC2_Random2	CBC2_Random2
	选择此项	选择此项	选择此项

后退 下一页

0% 100%

CBC2_Random3

情景#4/7个情景 **假设**当您正在经历一次发烧，但是您不确定您是否感染了新冠病毒。您决定寻求诊疗机构的帮助。您可能会面临以下几种关于诊疗服务的情景，某些情景可能并不符合现实情况，但也请您根据**假设**情况以及现有信息，从以下三个选项中选择您最满意的一种情景。

完成此问题后，您仍然可以随时单击“后退”按钮以随时返回此页面，并且可以随时更改答案。将鼠标移到疫苗属性的上方可以查看相关提示。

	诊疗服务 1	诊疗服务 2	都不选
医疗机构	私人医疗机构	电话咨询	都不选.
接诊人员	辅助护理人员	医生	
等待时间	0分钟	30分钟	
立即核酸检测	是	否	
诊断费用	150元	150元	
报销比率	60%	40%	
	CBC2_Random3<br>选择此项	CBC2_Random3<br>选择此项	CBC2_Random3<br>选择此项

后退 下一页

0% 100%

CBC2_Random4

情景#5/7个情景 **假设**当您正在经历一次发烧，但是您不确定您是否感染了新冠病毒。您决定寻求诊疗机构的帮助。您可能会面临以下几种关于诊疗服务的情景，某些情景可能并不符合现实情况，但也请您根据**假设**情况以及现有信息，从以下三个选项中选择您最满意的一种情景。

完成此问题后，您仍然可以随时单击“后退”按钮以随时返回此页面，并且可以随时更改答案。将鼠标移到疫苗属性的上方可以查看相关提示。

	诊疗服务 1	诊疗服务 2	都不选
医疗机构	私人医疗机构	网络咨询	都不选.
接诊人员	护士	医生	
等待时间	60分钟	30分钟	
立即核酸检测	否	是	
诊断费用	600元	450元	
报销比率	80%	60%	
	CBC2_Random4	CBC2_Random4	CBC2_Random4
	选择此项	选择此项	选择此项

后退 下一页

0% 100%

CBC2_Random5

情景#6/7个情景 **假设**当您正在经历一次发烧，但是您不确定您是否感染了新冠病毒。您决定寻求诊疗机构的帮助。您可能会面临以下几种关于诊疗服务的情景，某些情景可能并不符合现实情况，但也请您根据**假设**情况以及现有信息，从以下三个选项中选择您最满意的一种情景。

完成此问题后，您仍然可以随时单击“后退”按钮以随时返回此页面，并且可以随时更改答案。将鼠标移到疫苗属性的上方可以查看相关提示。

	诊疗服务 1	诊疗服务 2	都不选
医疗机构	医院急诊部门	医院发热门诊	都不选.
接诊人员	护士	护士	
等待时间	0分钟	75分钟	
立即核酸检测	是	是	
诊断费用	600元	300元	
报销比率	20%	0%	
	CBC2_Random5	CBC2_Random5	CBC2_Random5
	选择此项	选择此项	选择此项

后退 下一页

0% 100%

CBC2_Random6

情景#7/7个情景 **假设**当您正在经历一次发烧，但是您不确定您是否感染了新冠病毒。您决定寻求诊疗机构的帮助。您可能会面临以下几种关于诊疗服务的情景，某些情景可能并不符合现实情况，但也请您根据**假设**情况以及现有信息，从以下三个选项中选择您最满意的一种情景。

完成此问题后，您仍然可以随时单击“后退”按钮以随时返回此页面，并且可以随时更改答案。将鼠标移到疫苗属性的上方可以查看相关提示。

	诊疗服务 1	诊疗服务 2	都不选
医疗机构	医院发热门诊	私人医疗机构	都不选.
接诊人员	医生	辅助护理人员	
等待时间	15分钟	45分钟	
立即核酸检测	是	否	
诊断费用	150元	600元	
报销比率	100%	40%	
	CBC2_Random6<br>选择此项	CBC2_Random6<br>选择此项	CBC2_Random6<br>选择此项

后退 下一页

0% 100%

Q50

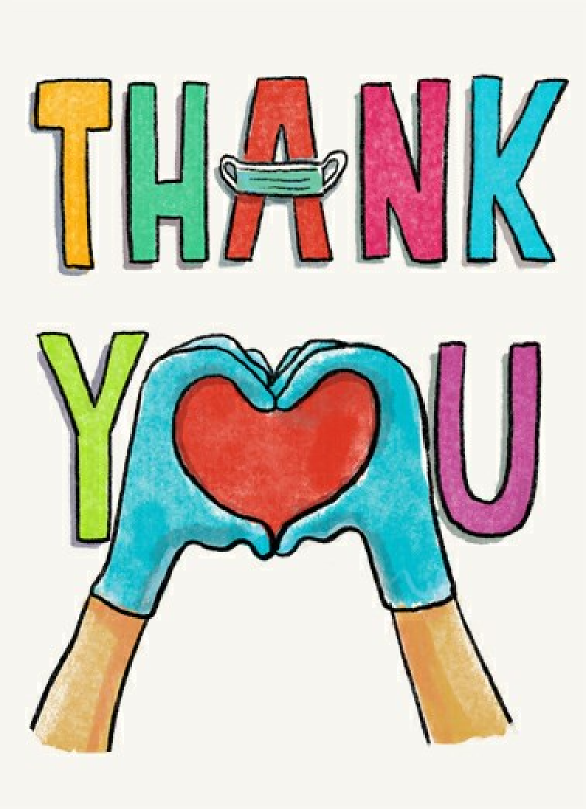

感谢您的支持！ 如果您对我们的研究感兴趣并希望深入参与我们的研究，可以发送电子邮件至：t.liu.10@student.rug.nl，我们将期待您的参与！ 我们将随机发送秘密奖励！

新冠病毒疫苗接种意愿研究组

[Script]

后退 下一页

0% 100%

end1

谢谢！

[Script]

0% 100%
