# Supplementary material for: Preferences for Attributes of Initial COVID-19 Diagnosis in the United States and China During the Pandemic: Discrete Choice Experiment With Propensity Score Matching
Source: JMIR Public Health Surveill. 2022 Aug 16;8(8):e37422. doi: 10.2196/37422 (PMC9384860; doi:10.2196/37422)
Supplement: Multimedia Appendix 3 [file publichealth_v8i8e37422_app3.docx]

**Supplementary information**

Table of Contents

Table S1. General results of the LCM. Data on latent class respondents’ preferences for diagnosis attributes in CHINA is reported…………...3

Table S2. General results of the LCM. Data on latent class respondents’ preferences for diagnosis attributes in the US is reported.....................5

Table S3. Fit Statistics From Latent Class Model of 2 to 5 Classes of China………………………………………....…………………………...7

Table S4. Fit Statistics From Latent Class Model of 2 to 5 Classes of US………………………………………....……………………………...8

Table S5. Interaction search for pre-PSM in China………………………………………....……………………………………………………..9

Table S6. Interaction search for PSM in China…………………………………………………………………………………………………...10

Table S7. Interaction search for pre-PSM in US……………………………………………………………………………………..…………...11

Table S8. Interaction search for PSM in US………………………………………………………...………………………………..………..….12

Table S9. Information of DCE task………..………………………………………………………...………………………………..………..….13

Table S10. Summary of test summaries…... ..……..………………………………………………………...…………………..………………..14

Table S11. The internal validity test results. ……………………………………………………...………………………………………………14

Table S12. Attribute Dominance test results(Unordered attributes are not included) …………..……………………………..…………………15

Table S13. Summary results. ……………………………………………………………………………………………………….……….……18

Figure S1. The flow chart of the Propensity Score Matching procedure (From January to March 2021). ………………………………………21

**Table S1. General results of the LCM. Data on latent class respondents’ preferences for diagnosis attributes in CHINA is reported.**

| Attributes and levels | | Class 1 (n=870) | | | Class 2 (n=270) | | | Class 3 (n=100) | | |
| --- | --- | --- | --- | --- | --- | --- | --- | --- | --- | --- |
|  | | Coefficient | Standard Error | *P* Value | Coefficient | Standard Error | *P* Value | Coefficient | Standard Error | *P* Value |
|  | |  |  |  |  |  |  |  |  |  |
| **Types of clinics** | | | | | | | | | | |
|  | Telephone consultation | -0.126 | 0.038 | .001 | -0.283 | 0.080 | .001 | -2.051 | 0.848 | .02 |
|  | Network diagnosis | -0.257 | 0.038 | <.001 | -0.486 | 0.083 | <.001 | -0.695 | 0.550 | .21 |
|  | Emergency | 0.276 | 0.038 | <.001 | 0.743 | 0.073 | <.001 | 1.076 | 0.348 | .003 |
|  | fever clinic | 0.232 | 0.038 | <.001 | 0.801 | 0.073 | <.001 | 0.673 | 0.357 | .06 |
|  | Private | -0.125 | 0.038 | .001 | -0.776 | 0.090 | <.001 | 0.998 | 0.350 | .005 |
| **Medical staff** | | | | | | | | | | |
|  | Nurse | -0.076 | 0.025 | .002 | -0.086 | 0.054 | .11 | -0.261 | 0.247 | .29 |
|  | Doctor | 0.225 | 0.025 | <.001 | 0.290 | 0.052 | <.001 | 0.536 | 0.218 | .02 |
|  | Paramedic | -0.148 | 0.025 | <.001 | -0.204 | 0.055 | <.001 | -0.275 | 0.256 | .29 |
| **Waiting time (minutes)** | | | | | | | | | | |
|  | 0 | 0.041 | 0.043 | .33 | 0.234 | 0.086 | .007 | 0.275 | 0.354 | .44 |
|  | 15 | 0.041 | 0.043 | .35 | 0.242 | 0.087 | .006 | 0.112 | 0.355 | .75 |
|  | 30 | -0.041 | 0.043 | .34 | 0.022 | 0.086 | .80 | 0.081 | 0.369 | .83 |
|  | 45 | -0.043 | 0.043 | .31 | -0.100 | 0.089 | .26 | -0.044 | 0.397 | .91 |
|  | 60 | -0.006 | 0.043 | .89 | -0.126 | 0.089 | .16 | -0.528 | 0.433 | .23 |
|  | 75 | 0.008 | 0.043 | .86 | -0.271 | 0.093 | .004 | 0.104 | 0.333 | .76 |
| **Immediate nucleic acid detection** | | | | | | | | | | |
|  | Yes | 0.121 | 0.015 | <.001 | 0.116 | 0.035 | .001 | 0.571 | 0.185 | .003 |
|  | No | -0.121 | 0.015 | <.001 | -0.116 | 0.035 | .001 | -0.571 | 0.185 | .003 |
| **Reimbursement rate(%)** | | | | | | | | | | |
|  | 0 | -0.277 | 0.043 | <.001 | 0.010 | 0.088 | .91 | 0.186 | 0.369 | .62 |
|  | 20 | -0.120 | 0.043 | .005 | -0.141 | 0.089 | .12 | -0.450 | 0.460 | .33 |
|  | 40 | -0.040 | 0.042 | .35 | -0.237 | 0.092 | .01 | -0.181 | 0.385 | .64 |
|  | 60 | -0.038 | 0.043 | .38 | 0.026 | 0.088 | .76 | -0.543 | 0.444 | .22 |
|  | 80 | 0.187 | 0.042 | <.001 | 0.136 | 0.087 | .12 | 0.428 | 0.334 | .20 |
|  | 100 | 0.287 | 0.043 | <.001 | 0.206 | 0.085 | .02 | 0.561 | 0.320 | .08 |
| **Cost ($)** | | | | | | | | | | |
|  | 0 | 0.093 | 0.038 | .01 | 0.184 | 0.077 | .02 | 1.146 | 0.277 | <.001 |
|  | 25 | 0.110 | 0.037 | .004 | 0.197 | 0.077 | .01 | -0.300 | 0.402 | .46 |
|  | 50 | -0.047 | 0.038 | .21 | -0.063 | 0.079 | .42 | 0.350 | 0.322 | .28 |
|  | 75 | -0.012 | 0.038 | .75 | -0.145 | 0.080 | .07 | -0.943 | 0.482 | .05 |
|  | 100 | -0.143 | 0.038 | <.001 | -0.173 | 0.079 | .03 | -0.253 | 0.391 | .52 |

**Table S2. General results of the LCM. Data on latent class respondents’ preferences for diagnosis attributes in the US is reported.**

| Attributes and levels | | Class 1 (n=269) | | | Class 2 (n=139) | | | Class 3 (n=832) | | |
| --- | --- | --- | --- | --- | --- | --- | --- | --- | --- | --- |
|  | | Coefficient | Standard Error | *P* Value | Coefficient | Standard Error | *P* Value | Coefficient | Standard Error | *P* Value |
|  | |  |  |  |  |  |  |  |  |  |
| **Types of clinics** | | | | | | | | | | |
|  | Telephone consultation | -0.176 | 0.082 | .03 | 0.897 | 0.372 | .02 | -0.073 | 0.039 | .06 |
|  | Network diagnosis | -0.313 | 0.086 | <.001 | 0.746 | 0.370 | .05 | -0.126 | 0.039 | .001 |
|  | Emergency | -0.162 | 0.083 | .05 | -0.808 | 0.633 | .20 | -0.057 | 0.039 | .14 |
|  | fever clinic | 0.188 | 0.079 | .02 | -1.127 | 0.670 | .01 | 0.123 | 0.039 | .002 |
|  | Private | 0.463 | 0.078 | <.001 | 0.291 | 0.405 | .47 | 0.132 | 0.038 | .001 |
| **Medical staff** | | | | | | | | | | |
|  | Nurse | -0.014 | 0.056 | .81 | 0.055 | 0.273 | .84 | 0.003 | 0.026 | .91 |
|  | Doctor | 0.309 | 0.055 | <.001 | 0.015 | 0.273 | .96 | 0.150 | 0.026 | <.001 |
|  | Paramedic | -0.296 | 0.058 | <.001 | -0.070 | 0.285 | .81 | -0.153 | 0.026 | <.001 |
| **Waiting time (minutes)** | | | | | | | | | | |
|  | 0 | 0.259 | 0.088 | .004 | 0.442 | 0.397 | .27 | 0.224 | 0.044 | <.001 |
|  | 15 | -0.005 | 0.092 | .96 | 0.107 | 0.419 | .80 | 0.098 | 0.044 | .03 |
|  | 30 | 0.179 | 0.089 | .04 | 0.353 | 0.454 | .44 | 0.041 | 0.044 | .35 |
|  | 45 | -0.207 | 0.094 | .03 | -0.146 | 0.419 | .73 | -0.070 | 0.044 | .12 |
|  | 60 | -0.004 | 0.092 | .96 | -0.541 | 0.529 | .31 | -0.121 | 0.044 | .006 |
|  | 75 | -0.222 | 0.094 | .02 | -0.214 | 0.460 | .64 | -0.173 | 0.044 | <.001 |
| **Immediate nucleic acid detection** | | | | | | | | | | |
|  | Yes | 0.324 | 0.037 | <.001 | 0.068 | 0.194 | .73 | 0.232 | 0.016 | <.001 |
|  | No | -0.324 | 0.037 | <.001 | -0.068 | 0.194 | .73 | -0.232 | 0.016 | <.001 |
| **Reimbursement rate(%)** | | | | | | | | | | |
|  | 0 | -0.244 | 0.094 | .01 | 0.384 | 0.435 | .38 | -0.261 | 0.044 | <.001 |
|  | 20 | -0.089 | 0.093 | .34 | -1.329 | 0.759 | .08 | -0.160 | 0.044 | <.001 |
|  | 40 | -0.139 | 0.093 | .14 | -0.819 | 0.659 | .22 | -0.017 | 0.044 | .69 |
|  | 60 | -0.016 | 0.091 | .86 | 0.805 | 0.386 | .04 | 0.030 | 0.044 | .49 |
|  | 80 | -0.028 | 0.091 | .76 | 0.845 | 0.389 | .03 | 0.115 | 0.043 | .008 |
|  | 100 | 0.517 | 0.087 | <.001 | 0.114 | 0.483 | .81 | 0.292 | 0.044 | <.001 |
| **Cost ($)** | | | | | | | | | | |
|  | 0 | 0.917 | 0.076 | <.001 | 4.776 | NA | .97 | 0.159 | 0.039 | <.001 |
|  | 25 | 0.289 | 0.077 | <.001 | 1.655 | NA | .99 | 0.119 | 0.039 | .002 |
|  | 50 | -0.125 | 0.081 | .13 | -11.255 | NA | .98 | 0.067 | 0.038 | .08 |
|  | 75 | -0.377 | 0.086 | <.001 | 2.171 | NA | .99 | -0.145 | 0.039 | <.001 |
|  | 100 | -0.704 | 0.093 | <.001 | 2.653 | NA | .98 | -0.200 | 0.039 | <.001 |

**Table S3. Fit Statistics From Latent Class Model of 2 to 5 Classes of China.**

| Number of Latent Classes | Replication | Log-likelihood | Pct Cert | AIC | CAIC | BIC | ABIC | Chi-Square | Relative Chi-Square |
| --- | --- | --- | --- | --- | --- | --- | --- | --- | --- |
| 2 | 3 | -6187.49284 | 22.62538 | 12464.98569 | 12820.15938 | 12775.15938 | 12632.15932 | 3618.61201 | 80.41360 |
| 3 | 2 | -6065.41009 | 24.15202 | 12266.82018 | 12803.52710 | 12735.52710 | 12519.43812 | 3862.77752 | 56.80555 |
| 4 | 2 | -6006.91900 | 24.88346 | 12195.83801 | 12914.07814 | 12823.07814 | 12533.90024 | 3979.75969 | 43.73362 |
| 5 | 4 | -5963.12308 | 25.43112 | 12154.24616 | 13054.01952 | 12940.01952 | 12577.75270 | 4067.35154 | 35.67852 |

**Table S4. Fit Statistics From Latent Class Model of 2 to 5 Classes of US.**

| Number of Latent Classes | Replication | Log-likelihood | Pct Cert | AIC | CAIC | BIC | ABIC | Chi-Square | Relative Chi-Square |
| --- | --- | --- | --- | --- | --- | --- | --- | --- | --- |
| 2 | 2 | -6253.35254 | 22.41997 | 12596.70508 | 12952.23592 | 12907.23592 | 12764.23576 | 3614.33164 | 80.31848 |
| 3 | 1 | -6091.84559 | 24.42365 | 12319.69118 | 12856.93779 | 12788.93779 | 12572.84866 | 3937.34554 | 57.90214 |
| 4 | 5 | -5994.67313 | 25.62919 | 12171.34626 | 12890.30862 | 12799.30862 | 12510.13053 | 4131.69046 | 45.40319 |
| 5 | 1 | -5917.97321 | 26.58074 | 12063.94641 | 12964.62454 | 12850.62454 | 12488.35747 | 4285.09031 | 37.58851 |

**Table S5. Interaction search for pre-PSM in China.**

| **Attributes** | **2LL *P*-Value for Interaction Effect** |
| --- | --- |
| Main Effects |  |
| + Clinics x Staff | .02 |
| + Clinics x Waiting time | .43 |
| + Clinics x COVID-19 tests | .31 |
| + Clinics x Diagnosis expenses | .26 |
| + Clinics x Reimbursement rate/Claims | .95 |
| + Staff x Waiting time | .26 |
| + Staff x COVID-19 tests | .64 |
| + Staff x Diagnosis expenses | .79 |
| + Staff x Reimbursement rate/Claims | .74 |
| + Waiting time x COVID-19 tests | .53 |
| + Waiting time x Diagnosis expenses | .63 |
| + Waiting time x Reimbursement rate/Claims | .63 |
| + COVID-19 tests x Diagnosis expenses | .88 |
| + COVID-19 tests x Reimbursement rate/Claims | .57 |
| + Diagnosis expenses x Reimbursement rate/Claims | <.001 |

**Table S6. Interaction search for PSM in China.**

| **Attributes** | **2LL *P*-Value for Interaction Effect** |
| --- | --- |
| Main Effects |  |
| + Clinics x Staff | .004 |
| + Clinics x Waiting time | .40 |
| + Clinics x COVID-19 tests | .55 |
| + Clinics x Diagnosis expenses | .73 |
| + Clinics x Reimbursement rate/Claims | .98 |
| + Staff x Waiting time | .85 |
| + Staff x COVID-19 tests | .64 |
| + Staff x Diagnosis expenses | .97 |
| + Staff x Reimbursement rate/Claims | .93 |
| + Waiting time x COVID-19 tests | .53 |
| + Waiting time x Diagnosis expenses | .85 |
| + Waiting time x Reimbursement rate/Claims | .48 |
| + COVID-19 tests x Diagnosis expenses | .06 |
| + COVID-19 tests x Reimbursement rate/Claims | .24 |
| + Diagnosis expenses x Reimbursement rate/Claims | .23 |

**Table S7. Interaction search for pre-PSM in US.**

| **Attributes** | **2LL *P*-Value for Interaction Effect** |
| --- | --- |
| Main Effects |  |
| + Clinics x Staff | .77 |
| + Clinics x Waiting time | .94 |
| + Clinics x COVID-19 tests | .90 |
| + Clinics x Diagnosis expenses | .72 |
| + Clinics x Reimbursement rate/Claims | .08 |
| + Staff x Waiting time | .10 |
| + Staff x COVID-19 tests | .33 |
| + Staff x Diagnosis expenses | .12 |
| + Staff x Reimbursement rate/Claims | .63 |
| + Waiting time x COVID-19 tests | .25 |
| + Waiting time x Diagnosis expenses | .45 |
| + Waiting time x Reimbursement rate/Claims | .85 |
| + COVID-19 tests x Diagnosis expenses | .85 |
| + COVID-19 tests x Reimbursement rate/Claims | .22 |
| + Diagnosis expenses x Reimbursement rate/Claims | <.001 |

**Table S8. Interaction search for PSM in US.**

| **Attributes** | **2LL *P*-Value for Interaction Effect** |
| --- | --- |
| Main Effects |  |
| + Clinics x Staff | .96 |
| + Clinics x Waiting time | .64 |
| + Clinics x COVID-19 tests | .93 |
| + Clinics x Diagnosis expenses | .63 |
| + Clinics x Reimbursement rate/Claims | .18 |
| + Staff x Waiting time | .50 |
| + Staff x COVID-19 tests | .55 |
| + Staff x Diagnosis expenses | .39 |
| + Staff x Reimbursement rate/Claims | .84 |
| + Waiting time x COVID-19 tests | .92 |
| + Waiting time x Diagnosis expenses | .11 |
| + Waiting time x Reimbursement rate/Claims | .80 |
| + COVID-19 tests x Diagnosis expenses | .25 |
| + COVID-19 tests x Reimbursement rate/Claims | .03 |
| + Diagnosis expenses x Reimbursement rate/Claims | .36 |

**Table S9.** Information of DCE task.

| Attributes | Levels |
| --- | --- |
|  |  |
| Types of clinics | Telephone consultation |
|  | Network consultation |
|  | Emergency Room |
|  | Fever clinic |
|  | Private clinic |
| Medical Staff | Doctor |
|  | Nurse |
|  | Paramedic |
| Waiting time(minutes) | 0 |
|  | 15 |
|  | 30 |
|  | 45 |
|  | 60 |
|  | 75 |
| COVID-19 Nucleic Acid Testing | Yes |
|  | NO |
| Reimbursement Ratio (%) | 0 |
|  | 20 |
|  | 40 |
|  | 60 |
|  | 80 |
|  | 100 |

**Table S10.** Summary of test summaries.

| Study feature | Range |
| --- | --- |
|  |  |
| Number of respondents | 8935 |
| Number of questions | 7 |
| Number of alternatives | 3 |
| Number of attributes | 6 |
| Minimum number of attributes levels | 2 |
| Maximum number of attribute levels | 6 |

**Table S11.** The internal validity test results.

| Test Type | % Failures |
| --- | --- |
|  |  |
| Stability | 0 |
| (repeated question) |  |
| Within-set dominated pairs | 2.4 |
|  |  |
| Across-set dominated pairs | 1.5 |
|  |  |
| Transitivity | 0 |

**Table S12.** Attribute Dominance test results(Unordered attributes are not included)

| Attribute | Number of Best Level Choices | Number of Respondents |
| --- | --- | --- |
|  |  |  |
| Waiting time | 0 | 6302 (70.5%) |
|  | 1 | 997 (11.2%) |
|  | 2 | 652 ( 7.3%) |
|  | 3 | 421 ( 4.7%) |
|  | 4 | 442 ( 4.9%) |
|  | 5 | 119 ( 1.3%) |
|  | 6 | 2 ( 0.0%) |
|  |  |  |
| COVID-19 Nucleic Acid Testing | 0 | 970 (10.9%) |
|  | 1 | 1268 (14.2%) |
|  | 2 | 1984 (22.2%) |
|  | 3 | 2023 (22.6%) |
|  | 4 | 1465 (16.4%) |
|  | 5 | 709 ( 7.9%) |
|  | 6 | 516 ( 5.8%) |
|  |  |  |
| Cost | 0 | 6281 (70.3%) |
|  | 1 | 1018 (11.4%) |
|  | 2 | 617 ( 6.9%) |
|  | 3 | 545 ( 6.1%) |
|  | 4 | 414 ( 4.6%) |
|  | 5 | 58 ( 0.6%) |
|  | 6 | 2 ( 0.0%) |
|  |  |  |
| Reimbursement Ratio | 0 | 948 (10.6%) |
|  | 1 | 1092 (12.2%) |
|  | 2 | 1830 (20.5%) |
|  | 3 | 2202 (24.6%) |
|  | 4 | 1642 (18.4%) |
|  | 5 | 871 ( 9.7%) |
|  | 6 | 350 ( 3.9%) |
|  |  |  |
| Alt A | 0 | 1125 (12.6%) |
|  | 1 | 1302 (14.6%) |
|  | 2 | 2027 (22.7%) |
|  | 3 | 2022 (22.6%) |
|  | 4 | 1402 (15.7%) |
|  | 5 | 574 ( 6.4%) |
|  | 6 | 483 ( 5.4%) |
|  |  |  |
| ALTB | 0 | 1346 (15.1%) |
|  | 1 | 1186 (13.3%) |
|  | 2 | 2048 (22.9%) |
|  | 3 | 2015 (22.6%) |
|  | 4 | 1472 (16.5%) |
|  | 5 | 633 ( 7.1%) |
|  | 6 | 235 ( 2.6%) |
|  |  |  |
| ALT C | 0 | 5911 (66.2%) |
|  | 1 | 896 (10.0%) |
|  | 2 | 653 ( 7.3%) |
|  | 3 | 426 ( 4.8%) |
|  | 4 | 285 ( 3.2%) |
|  | 5 | 182 ( 2.0%) |
|  | 6 | 582 ( 6.5%) |

**Table S13.** Summary results.

| **Number of best-level** | 0 | 1 | 2 | 3 | 4 | 5 | 6 |
| --- | --- | --- | --- | --- | --- | --- | --- |
| Waiting time | 70.5 | 11.2 | 7.3 | 4.7 | 4.9 | 1.3 | 0 |
| COVID-19 Nucleic Acid Testing | 10.9 | 14.2 | 22.2 | 22.6 | 16.4 | 7.9 | 5.8 |
| Cost | 70.3 | 11.4 | 6.9 | 6.1 | 4.6 | 0.6 | 0 |
| Reimbursement Ratio | 10.6 | 12.2 | 20.5 | 24.6 | 18.4 | 9.7 | 3.9 |
| Alt A | 12.6 | 14.6 | 22.7 | 22.6 | 15.7 | 6.4 | 5.4 |
| Alt B | 15.1 | 13.3 | 22.9 | 22.6 | 16.5 | 7.1 | 2.6 |
| Alt C | 66.2 | 10 | 7.3 | 4.8 | 3.2 | 2 | 6.5 |

| Attribute | Total Number of |
| --- | --- |
|  | Best-Level Choices |
| Waiting time | 5939 (9.9%) |
| COVID-19 Nucleic Acid Testing | 23806 (39.7%) |
| Cost | 5845 (9.7%) |
| Reimbursement Ratio | 24381 (40.7%) |
| Number of missing observations = 0 | |
| Number of respondents with short series =0 | |
| \| Test Type \| Ntests \| Nfail \| Nzero \| Fail1 \| Fail>1 \| Ntested \| Nresp \| \| --- \| --- \| --- \| --- \| --- \| --- \| --- \| --- \| \| Stability \| 0 \| 0 \| 8935 \| 0 \| 0 \| 8935 \| 8935 \| \| Bozo \| 858 \| 169 \| 8766 \| 169 \| 0 \| 8935 \| 8935 \| \| Monotonicity \| 9486 \| 262 \| 8798 \| 92 \| 45 \| 8935 \| 8935 \| \| Transitivity \| 0 \| 0 \| 8935 \| 0 \| 0 \| 8935 \| 8935 \| | |


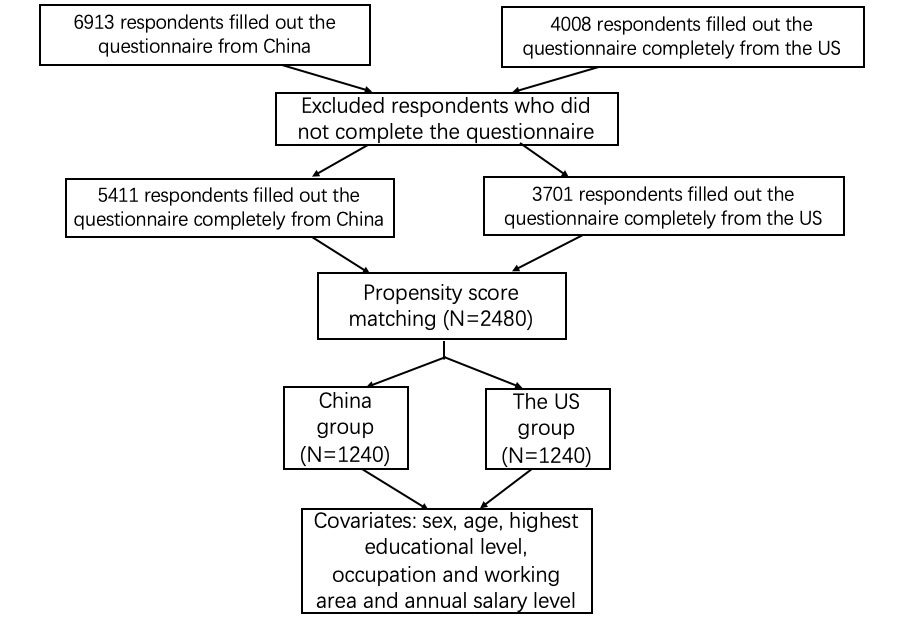


**Figure S1.** The flow chart of the Propensity Score Matching procedure (From January to March 2021)
